# Effect of burst spikes on linear and nonlinear signal transmission in spiking neurons

**DOI:** 10.1007/s10827-024-00883-1

**Published:** 2024-11-19

**Authors:** Maria Schlungbaum, Alexandra Barayeu, Jan Grewe, Jan Benda, Benjamin Lindner

**Affiliations:** 1 Bernstein Center for Computational Neuroscience Berlin, Philippstr. 13, Haus 2, 10115 Berlin, Germany; 2 Physics Department of Humboldt University Berlin, Newtonstr. 15, 12489 Berlin, Germany; 3 Neuroethology, Institute for Neurobiology of Eberhard Karls University Tübingen, Auf der Morgenstelle 28, 72076 Tübingen, Germany; 4 Bernstein Center for Computational Neuroscience Tübingen, Maria-von-Linden-Straße 6, 72076 Tübingen, Germany

**Keywords:** Stochastic neuron model, Bursting, Neural information transmission, Electrosensing

## Abstract

We study the impact of bursts on spike statistics and neural signal transmission. We propose a stochastic burst algorithm that is applied to a burst-free spike train and adds a random number of temporally-jittered burst spikes to each spike. This simple algorithm ignores any possible stimulus-dependence of bursting but allows to relate spectra and signal-transmission characteristics of burst-free and burst-endowed spike trains. By averaging over the various statistical ensembles, we find a frequency-dependent factor connecting the linear and also the second-order susceptibility of the spike trains with and without bursts. The relation between spectra is more complicated: besides a frequency-dependent multiplicative factor it also involves an additional frequency-dependent offset. We confirm these relations for the (burst-free) spike trains of a stochastic integrate-and-fire neuron and identify frequency ranges in which the transmission is boosted or diminished by bursting. We then consider bursty spike trains of electroreceptor afferents of weakly electric fish and approach the role of burst spikes as follows. We compare the spectral statistics of the bursty spike train to (i) that of a spike train with burst spikes removed and to (ii) that of the spike train in (i) endowed by bursts according to our algorithm. Significant spectral features are explained by our signal-independent burst algorithm, e.g. the burst-induced boosting of the nonlinear response. A difference is seen in the information transfer for the original bursty spike train and our burst-endowed spike train. Our algorithm is thus helpful to identify different effects of bursting.

## Introduction

Neurons encode information about sensory stimuli in sequences of action potentials. This process is strongly shaped by both the nonlinear neural dynamics of neurons that can lead to different kinds of spike patterns (Izhikevich, 2007) and the intrinsic stochasticity of neurons (Tuckwell, 1989). With respect to the latter aspect we note that several sources of noise lead to a certain degree of unreliability of the encoding process, limiting the transmission of information (Faisal et al., 2008). Many studies have focussed on the interplay of nonlinear dynamics of neurons, intrinsic noise sources, and time-dependent stimulation (Longtin, 1993; Greenwood et al., 2000; Lindner & Schimansky-Geier, 2001; Fourcaud & Brunel, 2002; Fourcaud-Trocme & Brunel, 2005; Longtin, 2009; Gai et al., 2010; Richardson & Swarbrick, 2010; McDonnell & Ward, 2011; Tchumatchenko et al., 2011; Alijani & Richardson, 2011; Doose et al., 2016; Voronenko & Lindner, 2017; Richardson, 2018; Schwalger, 2021; Franzen et al., 2023; Gowers & Richardson, 2023).

A striking feature seen in the spike trains of many cells is bursting: Action potentials occur in groups of narrowly spaced spikes led by a reference spike and extending over an (often random) number of burst spikes. There are many dynamical mechanisms for the generation of bursts already in deterministic (noise-free) models (Coombes & Bressloff, 2005; Izhikevich, 2007). It has been found by different types of input-output analyses that bursts may have a specific role in the encoding of information about particular kinds of stimuli (Oswald et al., 2007; Krahe & Gabbiani, 2004; Zeldenrust et al., 2018). However, we are far from a complete understanding of the role of bursts for neural signal transmission.

Here we want to contribute to a better understanding of the effects of bursting on signal transmission in a purely statistical manner. We are not interested in the nonlinear generation of bursts but choose to add bursts stochastically to a (burst-free) spike train. The advantage of this procedure is that we can relate input-output statistics of the bursting neuron to that of the non-bursting neuron and thus directly access certain effects of bursting on signal transmission both for the linear and the weakly nonlinear input-output signal transmission.

We deliberately do not take into account how the driving signal affects bursting but still obtain significant effects of the added bursts (and their variability, i.e. their stochastic features) on signal-transmission characteristics such as the linear and nonlinear susceptibilities and on the characteristics of the spontaneous activity, such as the power spectrum of the spike train. This was inspired by the approach chosen in the companion paper (Barayeu et al., 2024) for the analysis of bursting neurons, which we will here extend by systematically investigating the role of the number of burst spikes, its stochasticity, as well as the role of temporal jitter of the burst spikes.

In the following, we first introduce the spike train and signal transmission measures that will be used in this study. We then relate in a theoretical section these measures applied to the bursting spike train to that of the (non-bursting) reference spike train.

Using not yet precisely defined quantities (all statistics of interest are introduced in detail in the next section), we would like to give an anticipatory impression of the theoretical simplicity of our results. First of all, we find a multiplicative relation between the linear susceptibility with burst spikes, ${\chi }^{b}_{1}(\omega )$, and that without burst spikes, $\chi _{1}(\omega )$, of the form

I $$\begin{aligned} {\chi }^{b}_{1}(\omega ) = \chi _{1}(\omega ) f (\omega ) \end{aligned}$$

(see Eq. (23) and its derivation below). Here $f(\omega )$ is a frequency-dependent factor that is completely determined by our burst algorithm but independent of the reference spike train or any property of the neuron.

Similarly, the second-order susceptibilities (describing the weakly nonlinear response of the neuron) are as well connected by a purely multiplicative relation in terms of our function $f(\omega )$:

II $$\begin{aligned} {\chi }^{b}_{2}(\omega _{1},\omega _{2}) = \chi _{2}(\omega _{1},\omega _{2}) f(\omega _{1} + \omega _{2}) \end{aligned}$$

(see Eq. (29) and its derivation below).

For the power spectrum ${S}^{b}_{xx}(\omega )$, we find a somewhat more complicated relation with a multiplicative factor and an additional frequency-dependent offset $g(\omega )$ scaled by the firing rate $r_{0}$

III $$\begin{aligned} {S}^{b}_{xx}(\omega )= S_{xx}(\omega ) \left| f(\omega )\right| ^{2} + r_{0} g(\omega ) \end{aligned}$$

(see Eq. (44) and its derivation below); the offset function $g(\omega )$ is completely determined by the burst statistics but independent of the burst-free spectrum or other properties of the neuron. Although the structures of the relations Eq. (I)-(III) are simple, their derivations are not trivial, and we take our time to carefully outline how to arrive at these mathematically exact results.

We illustrate our analytical results for a simple stochastic leaky integrate-and-fire (LIF) model (non-bursting) to which we add bursts with algorithms of increasing complexity.

We finally apply our method to recordings of electro-sensory afferents in the weakly electric fish, the P-units, that *do* burst (Bastian, 1981a). Extending on the work in Barayeu et al. (2024), we first remove burst spikes but reintroduce them according to our most general algorithm. By comparison with the signal transmission properties of this surrogate burster to the original spike trains, we can identify aspects of the signal transmission that are simply related to adding stochastic burst spikes and those that are related to a more dynamical burst process that takes into account the neuron’s refractory period and, most importantly, the stimulus. Whereas deviations in the different response functions and power spectra between the original bursty spike trains and the surrogate bursters are only moderate, the differences are more pronounced in the spectral coherence function and the mutual information rate. The strongest differences are observed in a low-frequency band (below 50 Hz) and are most likely related to neglecting the spike frequency-adaptation mechanism in our statistical burst algorithm. Our strategy here (to develop a statistical bursting algorithm that captures key features of the data but clearly cannot capture certain other features) might be uncommon but thus provides useful insights.

Returning to the issue of neural information transmission, we note that the differences in the mutual information rate illustrate uniquely that real bursts in P-units increase the information transmission compared to the burst-free spike trains, whereas our (signal-unrelated) surrogate bursts can only lower the information transfer. Our results suggest that the physiology of P-units is suited to increase the information transmission by bursting.

## Measures of neural signal transmission

**Fig. 1:**
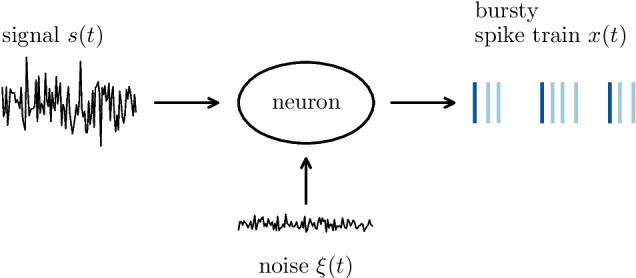
**Signal transmission by a spiking (bursting) neuron.** The neuron may fire packages of bursts (blue dashes on the right indicating the instances of spikes), i.e. each reference spike (dark blue) may be complemented by a (random) number of (randomly jittered) burst spikes (light blue dashed). We are interested in the effect of these additional spikes on the neural transmission of the time-dependent signal (on the left)

The basic problem addressed in our paper is sketched in Fig. 1: a time-dependent signal (left) is transmitted by a spike-generating neuron, that is in addition subject to a dynamical noise (bottom). The spike train generated (right) may be subdivided into tonic spikes (dark blue) and burst spikes (light blue). We are interested in the role of these burst spikes for the linear and nonlinear signal transmission by the neuron.

In the following, we recall briefly the spectral characteristics of spike trains and in particular measures of information transmission. We do not yet distinguish between bursting and non-bursting spike trains.

The mathematical representation of a spike train *x*(*t*) is given by a sum of Dirac delta functions

1 $$\begin{aligned} x(t) = \sum \limits _{k=1}^{N} \delta (t-t_k) \ , \end{aligned}$$

where *N* is the total number of spikes and $t_{k}$ are the time instances at which the spikes occur. In this work, we will consider Eq. (1) mainly in the Fourier domain since we are interested in the spectral statistics. The Fourier transform of the spike train over a time window [0, *T*] is given by

2 $$\begin{aligned} \tilde{x}(\omega ) = \int \limits _{0}^{T} \text {d}{t} \ e^{i\omega t} x(t) = \sum \limits _{k=1}^{N} e^{i\omega t_{k}} \ . \end{aligned}$$

We note that the choice of the time origin of the window (here $t=0$) is immaterial to the spectral analysis of a stationary time series. The variance of the different Fourier components of the spike train can be quantified by the spike-train power spectrum for $\omega \ne 0$

3 $$\begin{aligned} S_{xx}(\omega )&= \lim \limits _{T\rightarrow \infty } \frac{\left\langle \left| \tilde{x}(\omega )\right| ^{2} \right\rangle }{T}{= \lim \limits _{T\rightarrow \infty } \frac{\left\langle \tilde{x}(\omega )\tilde{x}^*(\omega ) \right\rangle }{T}} \ , \end{aligned}$$

where the asterisk denotes the complex conjugate. The brackets $\left\langle \cdot \right\rangle$ indicate an ensemble average, which means in this work either, for stochastic neuron models, different realizations of the intrinsic noise $\xi$ and the broadband stimulus *s*(*t*) or, for experiments, a trial average over recorded spike trains. Similarly, other stochastic time series can be characterized by their power spectrum, e.g. the Gaussian signal *s*(*t*) by its power spectrum $S_{ss}(\omega )$. For both the theoretical model and the experimental stimuli, bandpass-limited white Gaussian noise is used with a power spectrum

4 $$\begin{aligned} S_{ss}(\omega ) = {\left\{ \begin{array}{ll} 1, & \text {for } \left| \omega \right| \le \omega _{\textrm{cut}} \\ 0, & \text {for } \left| \omega \right| > \omega _{\textrm{cut}} \end{array}\right. } \ . \end{aligned}$$

Here, $\omega _{\textrm{cut}}$ is the cut-off frequency.

**Fig. 2:**
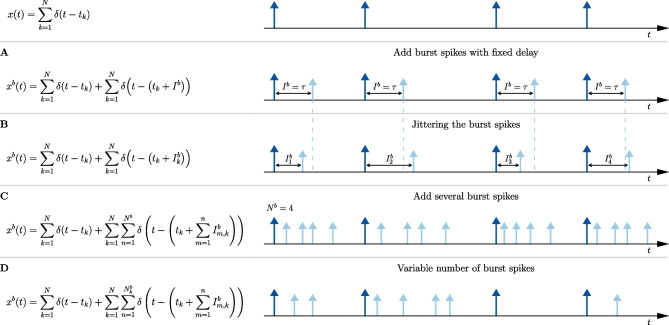
**Illustration of generating the burst spike train.** **Top panel** Reference spike train (spikes in dark blue). **A** Burst spike train with one burst spike (light blue): ${I}^{b} = \tau$. **B** Burst spike train with jittered IBIs ${I}^{b}_{k}$. **C** Burst spike train with ${N}^{b}$ burst spikes and jitter. **D** Burst spike train with different number of burst spikes for each reference spike. The case of having no burst spikes at a reference spike $t_{k}$ is also possible

The transfer of the signal can be captured by linear and nonlinear cross-correlations between input and output. Here we will consider specifically the second-order and third-order cross-spectra defined by

5 $$\begin{aligned} S_{xs}(\omega )&= \lim \limits _{T\rightarrow \infty } \frac{\left\langle \tilde{x}(\omega ) \tilde{s}^{*}(\omega ) \right\rangle }{T} \ , \end{aligned}$$

6 $$\begin{aligned} S_{xss}(\omega _{1},\omega _{2})&= \lim \limits _{T\rightarrow \infty } \frac{\left\langle \tilde{x}(\omega _{1}+\omega _{2}) \tilde{s}^{*}(\omega _{1}) \tilde{s}^{*}(\omega _{2}) \right\rangle }{T} \ . \end{aligned}$$

From these spectra, we can calculate the first- and second-order susceptibilities:

7 $$\begin{aligned} \chi _{1}(\omega )&= \frac{S_{xs}(\omega )}{S_{ss}(\omega )} \ , \end{aligned}$$

8 $$\begin{aligned} \chi _{2}(\omega _{1},\omega _{2})&= \frac{S_{xss}(\omega _{1},\omega _{2})}{2 S_{ss}(\omega _{1}) S_{ss}(\omega _{2})} \ . \end{aligned}$$

We recall that the weakly nonlinear mean response of the output (i.e. the instantaneous firing rate) to the input signal *s*(*t*) can be, to second order, expressed by

9 $$\begin{aligned} \left\langle x(t) \right\rangle&\approx \left\langle x(t) \right\rangle _{0} + \int \text {d}{t'}\ K_{1}(t-t') s(t') \nonumber \\&\quad + \int \text {d}{t_{1}} \int \text {d}{t_{2}} \ K_{2}(t-t_{1},t-t_{2}) s(t_{1})s(t_{2}) \ . \end{aligned}$$

The linear and nonlinear response functions $K_{1}$ and $K_{2}$ are the time versions of the above susceptibilities, i.e. the Fourier transforms with respect to one or two time arguments; the index 0 indicates the unperturbed state without a stimulus.

For very weak stimulation with Gaussian statistics, the information rate can be estimated by the lower bound formula (Rieke et al., 1993; Gabbiani, 1996; Rieke et al., 1996)

10 $$\begin{aligned} R_{\textrm{info}}= - \int \limits _{0}^{\omega _{\textrm{cut}}} \frac{\text {d}{\omega }}{2\pi } \ \log _{2}\left[ 1 - C(\omega )\right] \ , \end{aligned}$$

where the spectral *coherence function* is given in terms of the above introduced power and cross-spectra of output and stimulus:

11 $$\begin{aligned} C(\omega ) = \frac{\left| S_{xs}(\omega )\right| ^{2}}{S_{xx}(\omega ) S_{ss}(\omega )}. \end{aligned}$$

This is essentially the squared correlation coefficient between the Fourier coefficients of stimulus and output, i.e. at each frequency a number between zero (no linear correlations) and one (perfect correlation for the corresponding frequency components).

## Stochastic algorithm to add bursts to a spike train

Assuming that a non-bursting (reference) spike train *x*(*t*) is given, we may endow *x*(*t*) with burst spikes and thus create a burst spike train ${x}^{b}(t)$. Here we choose simple stochastic burst algorithms that still permit to analytically relate the spectral statistics of bursting and non-bursting spike trains. The algorithms are motivated by the kind of stochastic burst patterns seen in experimental data.

The most general form of the burst spike train is given by

12 $$\begin{aligned} {x}^{b}(t)&= x(t) + \sum \limits _{k=1}^{N} \sum \limits _{n=1}^{{N}^{b}_{k}} \delta \left( t - \left( t_{k} + \sum \limits _{m=1}^{n} {I}^{b}_{m,k} \right) \right) \nonumber \\&= x(t) + \sum \limits _{k=1}^{N} y_{k}(t) \ . \end{aligned}$$

Here $y_{k}(t)$ is a finite number of burst pulses added to the *k*-th reference spike. Different versions of the burst algorithm are illustrated in Fig. 2, and we explain the formula above in terms of those. In the simplest case (panel A), a single spike is added to each reference spike after a fixed delay $\tau$; i.e. the number of burst spikes ${N}^{b}_{k} = 1,\ \forall k$. The *intra-burst* interval (IBI), i.e. the interspike interval (ISI) within a burst, is given by the fixed delay ${I}^{b} = \tau$ yielding $y_{k}(t) = \delta \left( t - t_{k} - \tau \right)$.

As a first generalization, we include a temporal jitter (panel B); the number of burst spikes is still ${N}^{b}_{k} = 1$ and the IBIs ${I}^{b}_{k}$ are drawn independently from a distribution $\rho ({I}^{b})$ for each reference spike at $t_{k}$ (i.e. $y_{k}(t) = \delta ( t - t_{k} - {I}^{b}_{k})$). A simple example for an IBI distribution is a Gaussian with mean value $\langle {I}^{b}\rangle = \tau$ and standard deviation $\sigma$:

13 $$\begin{aligned} \rho \left( {I}^{b}\right)&= \frac{1}{\sqrt{2\pi \sigma ^{2}}} \exp \left[ -\frac{\left( \tau - {I}^{b}\right) ^{2}}{2\sigma ^{2}}\right] \ . \end{aligned}$$

To prevent overlapping IBI’s in case of adding multiple burst spikes, the standard deviation $\sigma$ of the IBI distribution is chosen such that the probability $\rho \left( {I}^{b}\right)$ will be sufficiently small for ${I}^{b} = \tau \pm \tau /2$, which is, for the Gaussian example, realized for $\sigma < \tau /4$.

In the next step (panel C), instead of a single burst spike we add a fixed number ${N}^{b}_{k}$ to each reference spike (${N}^{b}_{k} = 4,\ \forall k$ in panel C). Again, for each burst spike ${I}^{b}_{m,k}$ is drawn indepently and the corresponding burst-spike time is given by the sum of the reference-spike time $t_{k}$ and the sum of all previous IBIs within this burst. Put differently, we add a local *renewal spike train* $y_{k}(t)$ (a point process with statistically independent intervals between adjacent spikes) to each reference spike.

The last statistical feature incorporated (panel D) is to draw the burst spike number ${N}^{b}_{k}$ for each reference spike independently from a burst-spike distribution with probabilities $P_{j}$, $j\in \mathbb {N}$, which leads us to Eq. (12) in its most general form. We note that the case of having only a reference spike without a burst spike for a certain *k* is also possible by setting ${N}^{b}_{k} = 0$ (the corresponding *k*-th term in the second sum in Eq. (12) will then not contribute). In any case, here and in the following, the lower-case letters $n,m,\dots$ represent always summation indices; only the upper-case letters, *N* and *N* with index and/or superscripts, represent random (integer) variables.

We would like to point out that although we have added burst spikes as a local renewal process (Cox, 1962), the resulting burst spike trains are non-renewal processes. This is most obvious for the version shown in Fig. 2A and B, where a short interval is always followed by a long interval, yielding negative ISI correlations, but is also true in a more subtle manner for the other versions of the algorithm. Last but not least, we emphasize that we have made an implicit assumption of time-scale separation: the mean IBI multiplied with the mean number of burst spikes is typically much shorter than the ISI of the reference spike train such that the burst spikes of one reference spike do not fall into any other ISI than that following the reference spike.

## Relations between spectra of bursting and non-bursting spike trains

Given a set of reference spike trains $x_{i}(t)$, the corresponding set of burst spike trains ${x}^{b}_{i}(t)$, and a corresponding set of stimuli $s_{i}(t)$ for $i = 1, \ldots , N_{\textrm{r}}$, we would like to relate the spectral statistics introduced in Section 2 for the original (reference) spike train and for the burst spike train (burst spikes added according to the algorithm introduced in the preceding section).

We start with the burst-induced change in the linear susceptibility, then continue with the relation for the second-order susceptibility, and finally derive the relation between the spike train power spectra with and without bursting.

The linear susceptibility describes how a weak time-dependent stimulus affects the time-dependent firing rate (the instantaneous mean value of the spike train). The magnitude of the susceptibility $|\chi _1(\omega )|$ at a certain frequency can be interpreted as scaling the amplitude of the firing rate modulation, $r(t)=r_0+\varepsilon |\chi _1(\omega )| \sin (\omega t-\phi )$, in response to a very weak sinusoidal stimulus, $\varepsilon \sin (\omega t)$; of interest is here, whether stimulus-unrelated burst spikes may boost ($|{\chi }^{b}_{1}(\omega )|>|\chi _1(\omega )|$) or merely diminish ($|{\chi }^{b}_{1}(\omega )|<|\chi _1(\omega )|$) the linear response.

The next-order (nonlinear) response is characterized by the susceptibility ${\chi }^{b}_{2}(\omega _1,\omega _2)$ (or, for the reference spike train, $\chi _2(\omega _1,\omega _2)$) that depends on two frequency arguments and would describe the response up to second order in a small signal amplitude $\varepsilon$. Also here, we would like to know the effect of additional burst spikes on the response.

Last but not least, we also aim at the power spectrum for the case without stimulus (spontaneous activity). This statistics (in combination with the linear susceptibility) is useful to compute a lower bound on the neural information transmission (with and without burst spikes).

### Linear response function

First, we want to study the effect of burst spikes on the linear response function ${\chi }^{b}_{1}(\omega )$. Only the spike train is modified by the bursts (but not the signal) and hence only the cross-spectrum changes, $S_{xs} \rightarrow {S}^{b}_{xs}$, yielding

14 $$\begin{aligned} {\chi }^{b}_{1}(\omega )&= \frac{{S}^{b}_{xs}(\omega )}{S_{ss}(\omega )} \ . \end{aligned}$$

The Fourier transform of the burst spike train, Eq. (12), is given by

15 $$\begin{aligned} {\tilde{x}}^{b}(\omega )&= \tilde{x}(\omega ) + \sum \limits _{k=1}^{N} \sum \limits _{n=1}^{{N}^{b}_{k}} e^{i\omega \left( t_{k} + \sum \limits _{m=1}^{n} {I}^{b}_{m,k} \right) } \ , \end{aligned}$$

which inserted in Eq. (5) yields the burst cross-spectrum:

16 $$\begin{aligned} {S}^{b}_{xs}(\omega )&= \lim \limits _{T\rightarrow \infty } \frac{\left\langle \tilde{x}(\omega ) \tilde{s}^{*}(\omega ) \right\rangle }{T} + \nonumber \\&\quad \lim \limits _{T\rightarrow \infty } \frac{1}{T} \left\langle \sum \limits _{k=1}^{N} \sum \limits _{n=1}^{{N}^{b}_{k}} e^{i\omega \left( t_{k} + \sum \limits _{m=1}^{n} {I}^{b}_{m,k} \right) } \tilde{s}^{*}(\omega ) \right\rangle \ . \end{aligned}$$

Due to the additional randomness associated with the jittered IBIs and the burst-spike distribution, the brackets imply now not only an average over the intrinsic noise $\xi$ and the stochastic signal *s* but also over ${I}^{b}$ and ${N}^{b}$: $\left\langle \cdot \right\rangle = \left\langle \cdot \right\rangle _{\xi ,s,{I}^{b},{N}^{b}}$. The first term in Eq. (16) is the cross-spectrum between stimulus and reference spike train. This leaves only the calculation of the second term. First, we carry out the average with respect to the IBIs. Since the random numbers ${I}^{b}_{m,k}$ are drawn independently for each burst spike (they form the local renewal process $y_{k}(t)$), the average factorizes:

17 $$\begin{aligned} \left\langle e^{i\omega \sum \limits _{m=1}^{n} {I}^{b}_{m,k}} \right\rangle _{{I}^{b}}&= \prod \limits _{m=1}^{n} \left\langle e^{i\omega {I}^{b}_{m,k}} \right\rangle _{{I}^{b}} \nonumber \\&= \prod \limits _{m=1}^{n} \int \text {d}{{I}^{b}_{m,k}} \ e^{i\omega {I}^{b}_{m,k}} \rho \left( {I}^{b}_{m,k}\right) \nonumber \\&= \prod \limits _{m=1}^{n} \varphi _{{I}^{b}_{m,k}} (\omega ) = \varphi ^{n}_{{I}^{b}}(\omega ) \ . \end{aligned}$$

Here in the last line we used the definition of the *characteristic function* $\varphi (\omega )$, the Fourier transform of $\rho ({I}^{b}_{m,k})$, and that the ${I}^{b}_{m,k}$ are all drawn from the same distribution $\rho \left( {I}^{b}\right)$. For the example of the Gaussian, the characteristic function is well known:

18 $$\begin{aligned} \varphi _{\textrm{G}}(\omega )&=e^{i\omega \tau -\frac{\omega ^{2}\sigma ^{2}}{2}} \ . \end{aligned}$$

To average over the burst spikes we again use that the number ${N}^{b}_{k}$ of burst spikes is drawn independently for each reference spike $t_{k}$ in each realization (trial). For the total number of realizations $N_{\textrm{r}}$ we have for the *k*-th reference spike (assuming that it exists in every realization):

19 $$\begin{aligned} \left\langle \sum \limits _{n=1}^{{N}^{b}_{k}} \varphi _{{I}^{b}}^{n}(\omega ) \right\rangle _{{N}^{b}}&= \frac{1}{N_{\textrm{r}}} \Bigl [ \left( \varphi _{{I}^{b}} + \varphi _{{I}^{b}}^{2} + \ldots + \varphi _{{I}^{b}}^{{N}^{b}_{k,1}} \right) \nonumber \\&\qquad + \left( \varphi _{{I}^{b}} + \varphi _{{I}^{b}}^{2} + \ldots + \varphi _{{I}^{b}}^{{N}^{b}_{k,2}} \right) \Bigr . \nonumber \\&\qquad + \ldots \nonumber \\&\qquad + \Bigl . \left( \varphi _{{I}^{b}} + \varphi _{{I}^{b}}^{2} + \ldots + \varphi _{{I}^{b}}^{{N}^{b}_{k,N_{\textrm{r}}}} \right) \Bigr ] \nonumber \\&= \frac{1}{N_{\textrm{r}}} \left( p_{1}N_{\textrm{r}}\varphi _{{I}^{b}} + p_{2}N_{\textrm{r}}\varphi _{{I}^{b}}^{2} + \ldots \right) \nonumber \\&= \sum \limits _{n=1}^{\infty } p_{n} \varphi _{{I}^{b}}^{n}(\omega ) \ . \end{aligned}$$

Here we suppressed, for the ease of notation, the limit $N_{\textrm{r}} \rightarrow \infty$ that should be taken for a proper ensemble average. We arranged the terms to illustrate how the probability $p_{n}$ to have at least *n* burst spikes, emerges. The latter probability can be calculated from the probability $P_{j}$ to have exactly *j* burst spikes as follows:

20 $$\begin{aligned} p_{n} = \sum \limits _{j=n}^{\infty } P_{j} \ , \end{aligned}$$

and we may also easily invert this relation and write

21 $$\begin{aligned} P_{k} = p_{k}-p_{k+1} . \end{aligned}$$

The remaining averages for the second term in Eq. (16) are now the same as for the cross-spectrum $\left\langle \cdot \right\rangle = \left\langle \cdot \right\rangle _{\xi ,s}$, and we obtain for the burst cross-spectrum:

22 $$\begin{aligned} {S}^{b}_{xs}(\omega )&= S_{xs}(\omega ) + \lim \limits _{T\rightarrow \infty } \frac{1}{T} \times \nonumber \\&\quad \left\langle \sum \limits _{k=1}^{N} e^{i\omega t_{k}} \sum \limits _{n=1}^{\infty } p_{n} \varphi _{{I}^{b}}^{n}(\omega ) \tilde{s}^{*}(\omega ) \right\rangle \nonumber \\&= S_{xs}(\omega ) + \lim \limits _{T\rightarrow \infty } \frac{\left\langle \tilde{x}(\omega ) \tilde{s}^{*}(\omega ) \right\rangle }{T} \sum \limits _{n=1}^{\infty } p_{n} \varphi _{{I}^{b}}^{n}(\omega ) \nonumber \\&= S_{xs}(\omega ) \left( 1 + \sum \limits _{n=1}^{\infty } p_{n} \varphi _{{I}^{b}}^{n}(\omega ) \right) . \end{aligned}$$

Therefore, we find for the linear response function with burst spikes Eq. (14)

23 $$\begin{aligned} {\chi }^{b}_{1}(\omega )&= \chi _{1} (\omega ) \left( 1 + \sum \limits _{n=1}^{\infty } p_{n} \varphi _{{I}^{b}}^{n}(\omega ) \right) \nonumber \\&= \chi _{1}(\omega ) f (\omega ) , \end{aligned}$$

which is the linear response function given by Eq. (7) multiplied by a frequency-dependent factor. The latter depends on $\omega$ solely through the characteristic function, i.e. $f(\omega ) = F(\varphi (\omega ))$, where $F(\varphi )$ is a function of the characteristic function $\varphi$. Using the Gaussian approximation for the IBI distribution, the factor reads

24 $$\begin{aligned} f_{\textrm{G}}(\omega )&= 1 + \sum \limits _{n=1}^{\infty } p_{n} e^{n\left( i\omega \tau - \frac{1}{2}\omega ^{2}\sigma ^{2}\right) } , \end{aligned}$$

which assumes the form of a complex-valued damped oscillation with respect to the frequency argument $\omega$. The “frequency” of this undulation has the physical dimension of a time, corresponding to multiples of the delay $\tau$; the damping in turn is determined by the standard deviation of the jitter, $\sigma$.

We finally note that there is also a different interpretation for the terms in $f(\omega )$ that should also become apparent from our derivation above. For the nontrivial sum term we can write

$$\begin{aligned} \sum \limits _{n=1}^{\infty } p_{n} \varphi ^{n}_{{I}^{b}} (\omega )&= \sum \limits _{n=1}^{\infty } \sum _{j=n}^{\infty } P_{j} \int \limits _{0}^{\infty } \text {d}{T} \ e^{i\omega T} \rho _{n}(T) \\&= \sum \limits _{j=1}^{\infty } P_{j} \sum \limits _{n=1}^{j} \int \limits _{0}^{\infty } \text {d}{T}\ e^{i\omega T} \rho _{n}(T) \end{aligned}$$

In the first line of the above equation, we have used the *n*-th order interval density $\rho _{n}(T)$; in the second line we have exchanged the sums and obtained for a given burst count *n* a sum over all *n*-th order intervals giving us the probability to obtain *any* spike after the reference spike. The outer sum then averages this over all possible total numbers of burst spikes.

We can further simplify the right hand side by exploiting the fact that the probability density of spiking after the (arbitrarily chosen) *k*-th reference spike is the ensemble average of the renewal train $y_k(t_{k} + T)$, leading to

25 $$\begin{aligned} \sum \limits _{n=1}^{\infty } p_{n} \varphi ^{n}_{{I}^{b}} (\omega )&= \int \limits _{0}^{\infty } \text {d}{T}\ e^{i\omega T} \left\langle y_{k}(t_{k} + T) \right\rangle \nonumber \\&= \int \limits _{0}^{\infty } \text {d}{T}\ e^{i\omega T} m_{B}(T) = \widetilde{m}_{B}(\omega ) , \end{aligned}$$

where $m_{B}(T)$ is the conditional firing rate for a burst spike in the *k*-th burst at time $t_{k} + T$. In the last step of Eq. (25) it also becomes clear that for $\omega = 0$ the Fourier transform, turning into a pure integral over the conditional rate, yields the full mean number of burst spikes (for one burst and without counting the reference spike). Furthermore, the factor $f(\omega )$ can then be interpreted as the Fourier transform of

26 $$\begin{aligned} \delta (T) + m_{B}(T) , \end{aligned}$$

i.e. the conditional firing rate within a burst which includes (by the delta function) the reference spike itself.

### Nonlinear response function

Next, we would like to study the effect of burst spikes on the nonlinear response function Eq. (8):

27 $$\begin{aligned} {\chi }^{b}_{2}(\omega _{1}, \omega _{2})&= \frac{{S}^{b}_{xss}(\omega _{1},\omega _{2})}{2 S_{ss}(\omega _{1}) S_{ss}(\omega _{2})} . \end{aligned}$$

As before, the power spectrum of the signal $S_{ss}(\omega )$ is unaffected by the burst spikes, which leaves only the calculation of the third-order burst cross-spectrum ${S}^{b}_{xss}(\omega _{1}, \omega _{2})$. We obtain ${S}^{b}_{xss}(\omega _{1},\omega _{2})$ by inserting Eq. (15) now evaluated at $\omega \rightarrow \omega _{1} + \omega _{2}$ in Eq. (6):

28 $$\begin{aligned} {S}^{b}_{xss}(\omega _{1},\omega _{2})&= \lim \limits _{T\rightarrow \infty } \frac{\left\langle {\tilde{x}}^{b}(\omega _{1} + \omega _{2}) \tilde{s}^{*}(\omega _{1}) \tilde{s}^{*}(\omega _{2}) \right\rangle }{T} \nonumber \\&= S_{xss}(\omega _{1},\omega _{1}) \left( 1 + \sum \limits _{n=1}^{\infty } p_{n} \varphi _{{I}^{b}}^{n}(\omega _{1} + \omega _{2}) \right) . \end{aligned}$$

We can directly write down the result for the third-order burst cross-spectrum, because only the spike train is affected by the burst spikes, and all steps from the calculation of the second-order cross-spectrum Eqs. (16)-(22) apply in the same manner. For the nonlinear response function with burst spikes we then obtain:

29 $$\begin{aligned} {\chi }^{b}_{2}(\omega _{1},\omega _{2})&= \chi _{2}(\omega _{1}, \omega _{2}) \left( 1 + \sum \limits _{n=1}^{\infty } p_{n} \varphi _{{I}^{b}}^{n}(\omega _{1} + \omega _{2}) \right) \nonumber \\&= \chi _{2}(\omega _{1},\omega _{2}) f(\omega _{1} + \omega _{2}) \ , \end{aligned}$$

which is the nonlinear response function given by Eq. (8) multiplied with the same frequency-dependent factor as in Eq. (23), evaluated now at $\omega \rightarrow \omega _{1}+\omega _{2}$.

We note that for the Gaussian approximation the same applies as for the linear response: the factor $f_{\textrm{G}}(\omega _{1} + \omega _{2})$ introduces a damped oscillation into the nonlinear response function. Furthermore, the factor will be constant along the antidiagonal $\omega =\omega _{1} + \omega _{2}=\text {const}$, which suggests to consider a nonlinear response averaged over the anti-diagonal:

30 $$\begin{aligned} \mathbb {P}_{\!\!\chi _{2}} (\omega )\!&=\! {\left\{ \begin{array}{ll} \displaystyle \frac{\int \limits _{0}^{\omega } \text {d}{\omega _{1}} \left| \chi _{2} (\omega _{1}, \omega -\omega _{1}) \right| }{\int \limits _{0}^{\omega } \text {d}{\omega _{1}}}, & 0< \omega \le \omega _{\textrm{cut}} \\ \displaystyle \frac{\int \limits _{\omega - \omega _{\textrm{cut}}}^{\omega _{\textrm{cut}}}\!\!\!\!\!\! \text {d}{\omega _{1}} \left| \chi _{2} (\omega _{1}, \omega -\omega _{1}) \right| }{\int \limits _{\omega - \omega _{\textrm{cut}}}^{\omega _{\textrm{cut}}}\!\!\!\!\!\! \text {d}{\omega _{1}}}, & \omega _{\textrm{cut}}< \omega < 2\omega _{\textrm{cut}} \end{array}\right. } \end{aligned}$$

**Fig. 3:**
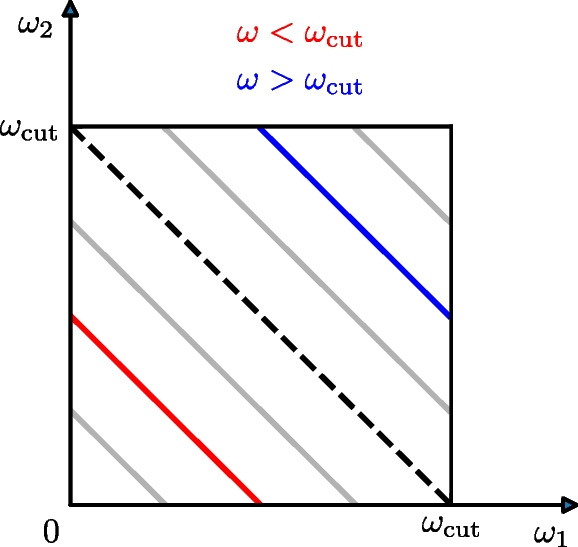
**Projection of the nonlinear response function.** The domain of the nonlinear response functions is limited by $\omega _{\textrm{cut}}$. We integrate over $\left| \chi _{2}(\omega _{1},\omega _{2}) \right|$ along the anti-diagonals $\omega = \omega _{1} + \omega _{2} =$ const and normalize these values by the length of the corresponding anti-diagonal. In the lower triangle we evaluate the projection for projection-frequencies $0 < \omega \le \omega _{\textrm{cut}}$ (red), and the upper triangle gives us the evaluation for the projection-frequencies $\omega _{\textrm{cut}}< \omega < 2\omega _{\textrm{cut}}$ (blue)

Because of the projection on the summed frequencies, this function is considered in the interval $\left( 0,2\omega _{\textrm{cut}}\right)$, i.e. up to twice the cut-off frequency $\omega _{\textrm{cut}}$. Figure 3 illustrates how Eq. (30) comes about.

### Spike train power spectrum

The effect of burst spikes on the power spectrum of the spike train is complicated due to the fact that it involves second-order statistics of the spike train itself. Inserting Eq. (15) in Eq. (3) yields

31 $$\begin{aligned} {S}^{b}_{xx}(\omega ) =&\lim \limits _{T\rightarrow \infty } \frac{\left\langle \tilde{x}(\omega )\tilde{x}^{*}(\omega ) \right\rangle }{T} \nonumber \\&+ \lim \limits _{T\rightarrow \infty } \frac{1}{T} \left[ \left\langle \tilde{x}(\omega ) \sum \limits _{k=1}^{N} \tilde{y}_{k}^{*}(\omega ) \right\rangle + \text {c.c.}\right] \nonumber \\&+ \lim \limits _{T\rightarrow \infty } \frac{1}{T} \left\langle \sum \limits _{k_{1}=1}^{N}\sum \limits _{k_{2}=1}^{N} \tilde{y}_{k_{1}}(\omega ) \tilde{y}_{k_{2}}^{*}(\omega ) \right\rangle \nonumber \\ =&\lim \limits _{T\rightarrow \infty } \frac{\left\langle \tilde{x}(\omega )\tilde{x}^{*}(\omega ) \right\rangle }{T} \nonumber \\&\hspace{-4em}+ \lim \limits _{T\rightarrow \infty } \frac{1}{T} \left[ \left\langle \tilde{x}(\omega ) \sum \limits _{k=1}^{N}\sum \limits _{n=1}^{{N}^{b}_{k}} e^{-i\omega \left( t_{k} + \sum \limits _{m=1}^{n} {I}^{b}_{m,k}\right) } \right\rangle + \text {c.c.}\right] \nonumber \\&\hspace{-4em}+ \lim \limits _{T\rightarrow \infty } \frac{1}{T} \left\langle \sum \limits _{k_{1}=1}^{N} \sum \limits _{n_{1} = 1}^{{N}^{b}_{k_{1}}} e^{i\omega \left( t_{k_{1}} + \sum \limits _{m_{1}=1}^{n_{1}} {I}^{b}_{m_{1},k_{1}}\right) } \times \right. \nonumber \\&\left. \hspace{2em} \sum \limits _{k_{2}=1}^{N} \sum \limits _{n_{2}=1}^{{N}^{b}_{k_{2}}} e^{-i\omega \left( t_{k_{2}} + \sum \limits _{m_{2}=1}^{n_{2}} {I}^{b}_{m_{2},k_{2}}\right) }\right\rangle \ . \end{aligned}$$

The brackets indicate an average over the intrinsic noise $\xi$, the IBIs ${I}^{b}$ and the burst-spike distribution $\left\langle \cdot \right\rangle = \left\langle \cdot \right\rangle _{\xi ,{I}^{b},{N}^{b}}$. The first term is the power spectrum of the reference spike train. The averages over the IBIs and burst-spike distribution in the second term can be calculated in the same way as for the second-order burst cross-spectrum in Eqs. (17) and (19). Therefore, we obtain for the first and second term in Eq. (31):

32 $$\begin{aligned} S_{xx}&(\omega ) \left( 1 + \sum \limits _{n=1}^{\infty } p_{n} \left[ \bigl ( \varphi _{{I}^{b}}(\omega ) \bigr )^{n} + \bigl ( \varphi _{{I}^{b}}^{*}(\omega ) \bigr )^{n} \right] \right) \nonumber \\&= S_{xx}(\omega ) \left( 1 + 2 \sum \limits _{n=1}^{\infty } p_{n}\, \text {Re}\{ \varphi _{{I}^{b}}^{n} (\omega ) \} \right) . \end{aligned}$$

To evaluate the third term, we distinguish between the terms of the sum referring to the same reference spike ($\sum _{k_{1}=k_{2}}$) or not ($\sum _{k_{1}\ne k_{2}}$). First, we want to focus on the second case: because we are referring to different reference spikes, $t_{k_{1}}$ and $t_{k_{2}}$, the random numbers ${I}^{b}_{m_{1},k_{1}}$ and ${I}^{b}_{m_{2},k_{2}}$ are independent. Therefore, the average over the IBI distribution can be calculated independently:

33 $$\begin{aligned} \left\langle e^{i\omega \sum \limits _{m_{1}=1}^{n_{1}} {I}^{b}_{m_{1},k_{1}}} \right\rangle _{{I}^{b}}&\left\langle e^{i\omega \sum \limits _{m_{2}=1}^{n_{2}} {I}^{b}_{m_{2},k_{2}}} \right\rangle _{{I}^{b}} \nonumber \\&= \bigl ( \varphi _{{I}^{b}} (\omega ) \bigr )^{n_{1}} \bigl ( \varphi _{{I}^{b}}^{*} (\omega ) \bigr )^{n_{2}} . \end{aligned}$$

Furthermore, this also allows us to compute the average over the burst-spike distribution independently (Eq. (19)):

34 $$\begin{aligned}&\left\langle \sum \limits _{n_{1}=1}^{{N}^{b}_{k_{1}}} \bigl ( \varphi _{{I}^{b}} (\omega ) \bigr )^{n_{1}} \right\rangle _{{N}^{b}} \left\langle \sum \limits _{n_{2}=1}^{{N}^{b}_{k_{2}}} \bigl ( \varphi _{{I}^{b}}^{*} (\omega ) \bigr )^{n_{2}} \right\rangle _{{N}^{b}} \nonumber \\&\qquad = \sum \limits _{n_{1}=1}^{\infty } p_{n_{1}} \bigl ( \varphi _{{I}^{b}} (\omega ) \bigr )^{n_{1}} \sum \limits _{n_{2}=1}^{\infty } p_{n_{2}} \bigl ( \varphi _{{I}^{b}}^{*} (\omega ) \bigr )^{n_{2}} . \end{aligned}$$

It remains to calculate the exponential containing the spike times:

35 $$\begin{aligned} \left\langle \sum \limits _{k_{1}\ne k_{2}}^{N} e^{i\omega (t_{k_{1}} - t_{k_{2}})} \right\rangle&= \left\langle \sum \limits _{k=1}^{N} (N-k) \left[ e^{i\omega T_{k}} + \text {c.c.}\right] \right\rangle \nonumber \\&= \left\langle \sum \limits _{k=1}^{N} (N - k) \bigl [ \tilde{p}_{k}(\omega ) + \tilde{p}_{k}^{*}(\omega ) \bigr ] \right\rangle \ . \end{aligned}$$

Here we have rewritten the differences of the spike times by means of the *k*-th order interval $T_{k} = t_{i+k} - t_{i}$ and assumed that the average over $\left\langle e^{i\omega T_{k}} \right\rangle$ does not depend on the spike-time index *i*; this is reflected by the suppression of the index in the notation of $T_{k}$ and by the prefactor $N-k$ of the number of identical terms appearing in the sum. Furthermore, in the second line we used the fact, that the average of the phase factor $\left\langle e^{i\omega T_{k}} \right\rangle$ for fixed *k* over different realizations of the intrinsic noise $\xi$ will result in the Fourier transform of the *k*-th order-interval density $\tilde{p}_{k}(\omega )$ (Holden, 1976). We keep the averaging brackets because the total spike count *N* is still a stochastic variable; in the limit $T\rightarrow \infty$, the prefactor $(N-k)/T$ approaches the firing rate $r_{0}$ and we may omit the averaging brackets. Combining all steps, Eqs. (33)-(35), in consideration of the last sum in Eq. (31), we obtain for the terms referring to different spike times:

36 $$\begin{aligned}&\lim \limits _{T\rightarrow \infty } \frac{1}{T}\left\langle \sum _{k_{1}\ne k_{2}} \sum \limits _{n_{1}=1}^{{N}^{b}_{k_{1}}} \sum \limits _{n_{2}=1}^{{N}^{b}_{k_{2}}} {A_{k_1,n_1}A^{*}_{k_2,n_2}} \right\rangle =\nonumber \\&r_{0}\!\!\sum _{k} \bigl [ \tilde{p}_{k}(\omega )\! +\! \tilde{p}_{k}^{*} (\omega ) \bigr ] \!\!\sum \limits _{n_{1}=1}^{\infty } \!\!p_{n_{1}} \!\bigl ( \varphi _{{I}^{b}} (\omega ) \bigr )^{n_{1}} \!\!\sum \limits _{n_{2}=1}^{\infty } \!\!p_{n_{2}} \!\bigl ( \varphi _{{I}^{b}}^{*} (\omega ) \bigr )^{n_{2}}\!\!, \end{aligned}$$

where $A_{k,n} = \exp \left[ i\omega \left( t_{k} + \sum _{m=1}^{n} {I}^{b}_{m,k}\right) \right]$. For the terms of the sum referring to the same reference spikes, we have to distinguish additionally between the terms of the sum referring to the same burst spikes ($\sum _{n_{1}=n_{2}}$) or not ($\sum _{n_{1}\ne n_{2}}$), yielding:

37 $$\begin{aligned} \left\langle N \sum _{n_{1}=n_{2}}^{{N}^{b}_{k_{1}}} 1 + N \underbrace{\sum \limits _{n_{1}\ne n_{2}}^{{N}^{b}_{k_{1}}} e^{i\omega \sum \limits _{m_{1}=1}^{n_{1}} {I}^{b}_{m_{1},k_{1}}} e^{-i\omega \sum \limits _{m_{2}=1}^{n_{2}} {I}^{b}_{m_{2},k_{1}}}}_{\mathcal {L}} \right\rangle \end{aligned}$$

The second sum $\mathcal {L}$ can be rewritten as follows:

38 $$\begin{aligned} \mathcal {L}= \left\langle \sum \limits _{n=1}^{{N}^{b}_{k_{1}}} \left( {N}^{b}_{k_{1}} - n \right) \left[ e^{i\omega \sum \limits _{m=1}^{n} {I}^{b}_{m,k_{1}}} + \text {c.c.}\right] \right\rangle _{{I}^{b}} \end{aligned}$$

Note, that we used here the fact, that the IBI’s are drawn from the same distribution and only the length of the sequence and not the explicit index $m_{1}$ or $m_{2}$ is important. Evaluating the averages $\left\langle \cdot \right\rangle _{{I}^{b},{N}^{b}}$ yields:

39 $$\begin{aligned}&\left\langle \sum \limits _{n=1}^{{N}^{b}_{k_{1}}} \left( {N}^{b}_{k_{1}} - n \right) \left[ e^{i\omega \sum \limits _{m=1}^{n} {I}^{b}_{m,k_{1}}} + \text {c.c.}\right] \right\rangle _{{I}^{b},{N}^{b}} \nonumber \\&= \left\langle 2\, \text {Re}\left\{ \sum \limits _{n=1}^{{N}^{b}_{k_{1}}} \left( {N}^{b}_{k_{1}} - n \right) \varphi _{{I}^{b}}^{n}(\omega )\right\} \right\rangle _{{N}^{b}} \nonumber \\&= \left\langle 2\, \text {Re}\left\{ \frac{\varphi _{{I}^{b}} \left( \varphi _{{I}^{b}}^{{N}^{b}_{k_{1}}} - {N}^{b}_{k_{1}} \varphi _{{I}^{b}} + {N}^{b}_{k_{1}} - 1 \right) }{\left( 1 - \varphi _{{I}^{b}} \right) ^{2}}\right\} \right\rangle _{{N}^{b}} \nonumber \\&= 2 \sum \limits _{n=1}^{\infty } P_{n}\, \text {Re}\left\{ \frac{\varphi _{{I}^{b}} \left( \varphi _{{I}^{b}}^{n} - n \varphi _{{I}^{b}} + n - 1 \right) }{\left( 1 - \varphi _{{I}^{b}} \right) ^{2}}\right\} \ , \end{aligned}$$

where we used in the second last step the result of the finite series

40 $$\begin{aligned} \sum \limits _{n=1}^{M} (M-n) a^{n} = \frac{a \left( a^{M} - aM + M - 1 \right) }{(1-a)^{2}} \ . \end{aligned}$$

As for the other terms, the ratio *N*/*T* approaches the firing rate in the limit of an infinite time window *T*. With the above, we obtain for the terms of the sum referring to the same spike times:

41 $$\begin{aligned} \lim \limits _{T\rightarrow \infty } \frac{1}{T}&\left\langle \sum \limits _{k_{1}=k_{2}} \sum \limits _{n_{1}=1}^{{N}^{b}_{k_{1}}} \sum \limits _{n_{2}=1}^{{N}^{b}_{k_{2}}}{A_{k_1,n_1}A^{*}_{k_2,n_2}} \right\rangle = r_{0} \sum \limits _{n=1}^{\infty } p_{n} + \nonumber \\&\quad 2 r_{0} \sum \limits _{n=1}^{\infty } P_{n}\, \text {Re}\left\{ \frac{\varphi _{{I}^{b}} \left( \varphi _{{I}^{b}}^{n} - n \varphi _{{I}^{b}} + n - 1 \right) }{\left( 1 - \varphi _{{I}^{b}} \right) ^{2}}\right\} \ . \end{aligned}$$

Using the general result for a stationary spike train (Holden, 1976)

42 $$\begin{aligned} S_{xx} (\omega ) = r_{0} \left( 1 + \sum \limits _{k} \bigl [ \tilde{p}_{k}(\omega ) + \tilde{p}_{k}^{*} (\omega ) \bigr ] \right) \ , \end{aligned}$$

and our results in Eqs. (32), (36) and (41), we obtain for the burst-spike-train power spectrum:

43 $$\begin{aligned} {S}^{b}_{xx}(\omega ) =&S_{xx} (\omega ) \left[ 1 + 2 \sum \limits _{n=1}^{\infty } p_{n}\, \text {Re}\left\{ \varphi _{{I}^{b}}^{n} (\omega ) \right\} + \right. \nonumber \\&\left. \sum \limits _{n_{1}=1}^{\infty } p_{n_{1}} \bigl ( \varphi _{{I}^{b}} (\omega ) \bigr )^{n_{1}} \sum \limits _{n_{2}=1}^{\infty } p_{n_{2}} \bigl ( \varphi _{{I}^{b}}^{*} (\omega ) \bigr )^{n_{2}} \right] \nonumber \\&+ r_{0} \left[ \sum \limits _{n=1}^{\infty } p_{n} \!-\! \sum \limits _{n_{1}=1}^{\infty } p_{n_{1}} \bigl ( \varphi _{{I}^{b}} (\omega ) \bigr )^{n_{1}} \sum \limits _{n_{2}=1}^{\infty } p_{n_{2}} \bigl ( \varphi _{{I}^{b}}^{*} (\omega ) \bigr )^{n_{2}} \right. \nonumber \\&+ \left. 2 \sum \limits _{n=1}^{\infty } P_{n}\, \text {Re}\left\{ \frac{\varphi _{{I}^{b}} \left( \varphi _{{I}^{b}}^{n} - n \varphi _{{I}^{b}} + n - 1 \right) }{\left( 1 - \varphi _{{I}^{b}} \right) ^{2}}\right\} \right] \end{aligned}$$

This can be further simplified to yield

44 $$\begin{aligned}&{S}^{b}_{xx}(\omega )= S_{xx}(\omega ) \left| f(\omega ) \right| ^{2} + \nonumber \\&r_{0} \left[ \sum \limits _{n=1}^{\infty } p_{n} \left( 1 + 2\, \text {Re}\left\{ \frac{\varphi _{{I}^{b}} \left( 1 - \varphi _{{I}^{b}}^{n-1} \right) }{1 - \varphi _{{I}^{b}}}\right\} \right) - \left| f(\omega ) - 1 \right| ^{2} \right] \nonumber \\&= S_{xx}(\omega ) \left| f(\omega )\right| ^{2} + r_{0} g(\omega ) \ . \end{aligned}$$

Here we have used Eq. (21), performed a few of algebraic manipulations, and expressed the sum term with the factor $f(\omega )$ via

$$\begin{aligned} \sum \limits _{n_{1}=1}^{\infty } p_{n_{1}} \bigl ( \varphi _{{I}^{b}} (\omega ) \bigr )^{n_{1}}&= f(\omega ) - 1 \ . \end{aligned}$$

Unlike the response functions we do not obtain in Eq. (44) the burst-spike-train power spectrum by a pure product of the reference spike-train spectrum and a frequency-dependent factor. Besides the original spectrum (first line) being multiplied with the squared absolute value of the factor introduced in Eq. (23), we have now also an additional term that includes the firing rate $r_{0}$ multiplied by a function $g(\omega )$ of the burst characteristics.

When we inspect the high-frequency limit of the spectrum, it is useful to know the limits for the characteristic function $\varphi _{{I}^{b}}(\omega )$ and the factor $f(\omega )$. In the case of jittered IBI’s with a smooth probability density, we can assume that

45 $$\begin{aligned} \lim \limits _{\omega \rightarrow \infty } \varphi _{{I}^{b}}(\omega )&= 0 \quad \Rightarrow \quad \lim \limits _{\omega \rightarrow \infty } f(\omega ) = 1 \ , \end{aligned}$$

i.e. the probability density does not change in an infinitely fast manner, and hence its Fourier transform decays for very high frequencies.

The additive term in Eq. (44) ensures the saturation of ${S}^{b}_{xx}$ in the large frequency limit at an increased firing rate

46 $$\begin{aligned} \lim \limits _{\omega \rightarrow \infty } {S}^{b}_{xx} (\omega )&= \lim \limits _{\omega \rightarrow \infty } S_{xx}(\omega ) + r_{0} \sum \limits _{n=1}^{\infty } p_{n} \nonumber \\&= r_{0} \left( 1 + \left\langle {N}^{b} \right\rangle \right) = {r}^{b}_{0} \ , \end{aligned}$$

which is then just the burst firing rate ${r}^{b}_{0}$. Notably, we recognize from Eq. (44) that without a jitter (when $\varphi _{\textrm{G}}(\omega ) = e^{i\omega \tau }$) the burst-spike-train power spectrum will not saturate at ${r}^{b}_{0}$ but oscillate around this value.

From our derivation it is also evident that the additive term in Eq. (44) can never be negative,

47 $$\begin{aligned} g(\omega )&= \sum \limits _{n=1}^{\infty } p_{n} \left( 1 + 2\,\text {Re}\left\{ \frac{\varphi _{{I}^{b}} \left( 1 - \varphi _{{I}^{b}}^{n-1} \right) }{1 - \varphi _{{I}^{b}}}\right\} \right) \nonumber \\&\quad - \left| f(\omega ) - 1 \right| ^{2} \nonumber \\&\ge 0 \ . \end{aligned}$$

This inequality is not obvious but can be understood by considering the burst $y_{k}(t) = \sum \limits _{i=1}^{{N}^{b}_{k}} \delta (t-t_{k,i})$, i.e. the finite (stochastic) number of burst spikes added to the *k*-th spike. Then its mean value is given by

48 $$\begin{aligned} \left\langle y_{k}(t) \right\rangle&= m_{B}(t - t_{k}) \ , \quad t > t_{k} \ . \end{aligned}$$

The (one-sided) Fourier transform of this function is given by $f(\omega ) - 1$. From our derivation it has become clear that the infinite sum in the first line of Eq. (47) is equal to the second moment of $\tilde{y}_{k}(\omega )$, cf. the first line in Eq. (31) and in particular the terms in the last double sum with $k_{1}=k_{2}$. Hence, the left hand side of Eq. (47) can be regarded as a variance

49 $$\begin{aligned} g(\omega )&= \left\langle \tilde{y}_{k}(\omega ) \tilde{y}_{k}^{*}(\omega ) \right\rangle - \left\langle \tilde{y}_{k}(\omega ) \right\rangle \left\langle \tilde{y}_{k}^{*}(\omega ) \right\rangle \ge 0 \ , \end{aligned}$$

which cannot be negative.

### Coherence function

The seemingly exotic non-negativity of the additional term in the power spectrum is consistent with the insight that by adding burst spikes in a signal-unrelated manner to a spike train, we can only degrade the information that the spike train carries about the stimulus. At the level of a linear approximation this becomes apparent in terms of the coherence function between burst spike train and stimulus

50 $$\begin{aligned} {C}^{b}(\omega )&= \frac{\left| {S}^{b}_{xs}(\omega ) \right| ^{2}}{S_{ss}(\omega ) {S}^{b}_{xx}(\omega )} = \frac{\left| {\chi }^{b}_{1}(\omega ) \right| ^{2} S_{ss}(\omega )}{{S}^{b}_{xx}(\omega )} \nonumber \\&= \frac{\left| \chi _{1}(\omega ) \right| ^{2} \left| f(\omega ) \right| ^{2} S_{ss}(\omega )}{S_{xx} \left| f(\omega ) \right| ^{2} + r_{0} g(\omega )} \nonumber \\&= \frac{S_{xx}(\omega )}{S_{xx}(\omega ) + r_{0} g(\omega )/\left| f(\omega ) \right| ^{2}} C(\omega ) \ , \end{aligned}$$

where $C(\omega )$ is the coherence between the stimulus and the reference spike train. From the structure of the prefactor it is clear that as long as $g(\omega ) \ge 0$, which was shown in the relations Eqs. (47)-(49), we have

51 $$\begin{aligned} {C}^{b}(\omega ) \le C(\omega ) \ , \end{aligned}$$

i.e. we never increase the correlation coefficient between input and output by adding burst spikes to the spike train in a signal-unrelated manner. Likewise, we can conclude that by adding burst spikes in this way we cannot increase the lower bound of the mutual information rate, Eq. (10), which is a monotonic function of the coherence function. We will discuss these information-theoretic measures for the P-units in Section 6.3.

## Testing the relations for a stochastic integrate-and-fire neuron

### Neuron model driven by bandpass-limited noise

To test our derived formulas, we consider the LIF neuron as an example for a non-bursting and stochastically spiking neuron model (Holden, 1976; Tuckwell, 1989; Burkitt, 2006; Vilela & Lindner, 2009) to which we can apply our bursting algorithm. The dynamics of the *i*-th realization (trial), $i = 1, \ldots , N_{\textrm{r}}$, is given by

52 $$\begin{aligned} \dot{v}_{i}(t)&= -v_{i}(t) + \mu + \sqrt{2D}\xi _{i}(t) \ . \end{aligned}$$

$v_{i}(t)$ denotes the membrane voltage, $\mu$ is the mean input current, and time is measured in multiples of the membrane time constant (i.e. we use a non-dimensional time variable). Additionally, we apply a fire-and-reset rule: whenever $v_{i}(t)$ hits a threshold $v_{T}$, an output spike time $t_{k}$ is registered and the voltage is reset to $v_{R}$. The sum of delta functions at the so determined spike times, $x(t) = \sum _{k} \delta (t-t_{k})$, constitutes the main output of the LIF neuron. The voltage is given in multiples of the threshold-reset difference, and we set $v_{R} = 0$ and $v_{T} = 1$.

For the noise process in Eq. (52) we will use a bandpass-limited white Gaussian noise with a power spectrum given in Eq. (4) determined by a cut-off frequency that is much higher than the firing rate of the neuron; here the effect of the noise on the LIF dynamics is close to that of a true white Gaussian noise with a delta correlation function, a version of the LIF model that has been studied thoroughly in the literature (Holden, 1976; Ricciardi, 1977; Tuckwell, 1989; Lindner, 2002; Fourcaud & Brunel, 2002; Burkitt, 2006).

We aim for a measurement scheme to probe the whole frequency range of the system. In our simulation, we know the exact values of the noise and can regard a fraction of this stochastic process as an input signal:

53 $$\begin{aligned} \xi _{i}(t)&= \sqrt{1-c} \xi _{i,n}(t) + \sqrt{c} \xi _{i,s}(t) \ , \end{aligned}$$

where $\xi _{i,n}$ denotes the intrinsic background part and $\xi _{i,s}$ is the signal, and both parts are Gaussian distributed and statistically independent of each other (such a subdivision is possible for Gaussian signals). The parameter *c* is a scaling factor indicating how much of the full noise $\xi _{i}$ is considered to be the input signal.

As a consequence of the Furutsu-Novikov theorem (Novikov, 1965) and its generalization to higher-order response functions (Egerland, 2021), the equations for the first- and second-order susceptibility Eqs. (7) and (8) read now:

54 $$\begin{aligned} \chi _{1} (\omega ; D)&= \frac{S_{xs}(\omega )}{2Dc} \ , \end{aligned}$$

55 $$\begin{aligned} \chi _{2} (\omega _{1}, \omega _{2}; D)&= \frac{S_{xss}(\omega _{1}, \omega _{2})}{2(2Dc)^{2}} \ . \end{aligned}$$

Here, we have indicated the dependence of the response functions on the total noise level - specifically, we want to emphasize that the response functions do not depend on the splitting parameter *c* (for a discussion of this problem, see Vilela and Lindner (2009)). We furthermore note that the power spectrum of the spike train is independent of *c* as well.

In the following, we inspect how the response functions and the spectrum change upon addition of burst spikes according to our algorithm and to the derived relations. We choose one specific set of system parameters, $\mu = 0.9$, $D=0.005$, corresponding to the excitable regime of the LIF neuron with an intermediate noise level. Here, the stationary firing rate is $r_{0} = 0.12$ corresponding to a mean ISI of $\left\langle I \right\rangle = 8.33$. For the IBI distribution we choose a Gaussian with mean IBI $\tau = 0.5$, which is much smaller than the mean ISI, and we set the standard deviation either $\sigma = 0.0$ or $\sigma = 0.13$.

### Linear response function

**Fig. 4:**
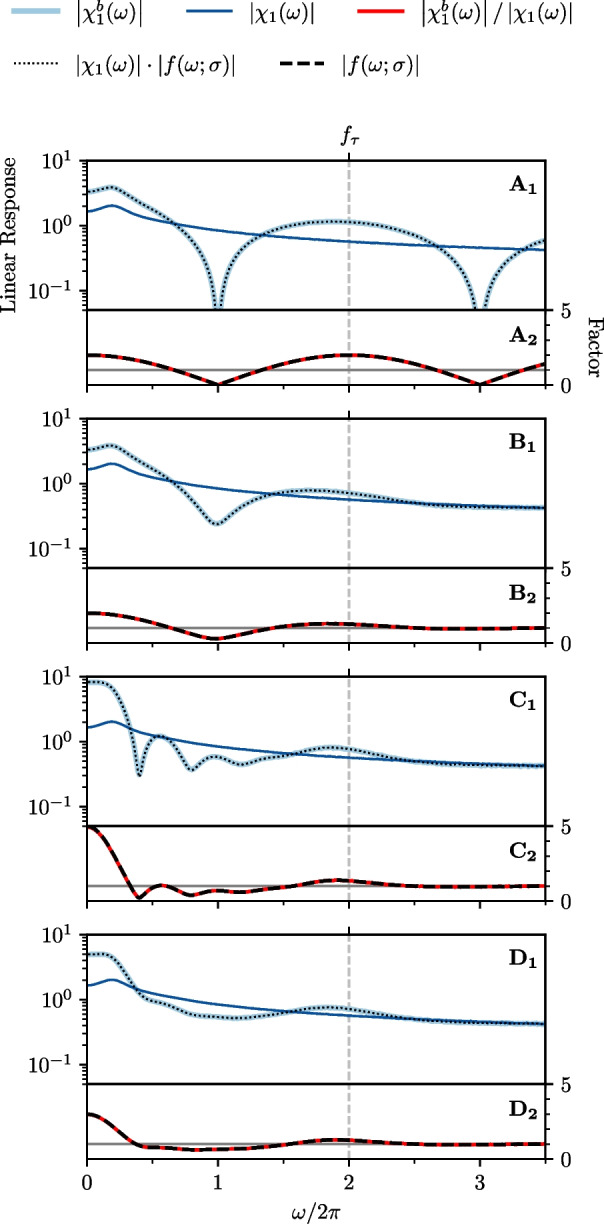
**LIF neuron: Effect of burst spikes on the linear response function. A-D** same as in Fig. 2. Upper panels [1]: Numerical measurement of the susceptibility (dark blue) and burst susceptibility (light blue) compared to the theoretical prediction of the burst susceptibility (black dotted line), Eq. (23). Lower panels [2]: Numerical (red) and analytical (black dashed line) estimation of the factor $f(\omega ;\sigma )$. In all panels, only the absolute values are plotted. Model parameters: $\mu =0.9$, $D=0.005$, $\tau =0.5$, $\sigma = 0.0$ in **A** and $\sigma = 0.13$ in **B-D**. **D** Number of burst spikes is drawn from a uniform distribution: ${N}^{b} \in \left\{ 0,1,2,3,4\right\}$

We start with the linear response function and show the results in Fig. 4. The panels A-D correspond to the different burst-algorithms depicted in Fig. 2. In the respective upper panel (index 1) we plot the numerical estimation of the absolute values of the susceptibility $\left| \chi _{1}(\omega ) \right|$ (dark blue), the burst-susceptibility $\left| {\chi }^{b}_{1}(\omega ) \right|$ (light blue) and the theoretical prediction of the absolute value of the burst-susceptibility (black dotted line) given by Eq. (23). In the lower panel (index 2) we show the absolute value of the numerical (red) and analytical (black dashed line) evaluation of the factor $\left| f_{\textrm{G}}(\omega ) \right|$, Eq. (24), for the corresponding burst algorithms.

First of all, we find an excellent agreement for our numerical and analytical results. Furthermore, the addition of burst spikes causes a periodic modulation of the linear response function. Focusing first on the case of one burst spike with no temporal jitter, i.e. the standard deviation of the jitter distribution is zero, $\sigma = 0$ (panel A), this modulation with respect to frequency has the period $f_{\tau } = 1/\tau = 2.0$ (which has the dimension of a frequency and not of a time). The modulating factor is $f_{\textrm{G}}(\omega ) = 1 + e^{i\omega \tau }$, the absolute value of which yields an undamped oscillation, $\left| f_{\textrm{G}}(\omega ) \right| = \sqrt{2 + 2\cos \,({\omega \tau })}$ (Fig. 4A$_{2}$). By adding one burst spike without a jitter we can double the absolute value of the linear response function for each driving frequency equal to a multiple of $f_{\tau }$. A similar effect could be achieved by doubling the weight of each output spike, $x(t)\rightarrow 2x(t)$.

Including the jitter leads to a damping of the oscillation that is determined by $\sigma$. In Fig. 4B we choose $\sigma = 0.13$, which yields a strong suppression of the oscillation shortly after the first cycle. For arbitrary values of $\sigma$ and in accordance with the general limit case Eq. (45), we find in the high-frequency limit $\lim \limits _{\omega \rightarrow \infty } f_{\textrm{G}}(\omega ) = 1$, which means that the burst spikes have no effect on the linear response function for $\omega \rightarrow \infty$ when the burst-spike times are jittered.

When we increase the number of burst spikes (${N}^{b}_{k} = 4$, $\forall k$ in panel C), we obtain subharmonic modes corresponding to multiples of the delay time $\tau$ and specifically depending on ${N}^{b}_{k}$. The high-frequent modulation hinges on the fact that the number of burst spikes is always the same. If we randomize the number of burst spikes (uniform distribution over ${N}^{b}_{k} \in \left\{ 0,1,2,3,4\right\}$ in panel D), the ondulation of the susceptibility is strongly diminished. The remaining effect of the burst spikes in this most general version of our algorithm is a boost of the linear response at very low frequencies and around the frequency $f_{\tau }$ corresponding to the delay time and a reduction between these two frequency ranges. Specifically, for the boost at low frequencies, we find

$$\begin{aligned} \lim \limits _{\omega \rightarrow 0} f_{\textrm{G}}(\omega )&= 1 + \sum \limits _{n=1}^{\infty } p_{n} = 1 + \left\langle {N}^{b}_{k} \right\rangle \ . \end{aligned}$$

Therefore, the boosting effect of the burst spikes is here increased by a larger mean number of burst spikes. In particular, the amplification is larger in panel C than in D because the mean values $\left\langle {N}^{b}_{k} \right\rangle$ differ.

**Fig. 5:**
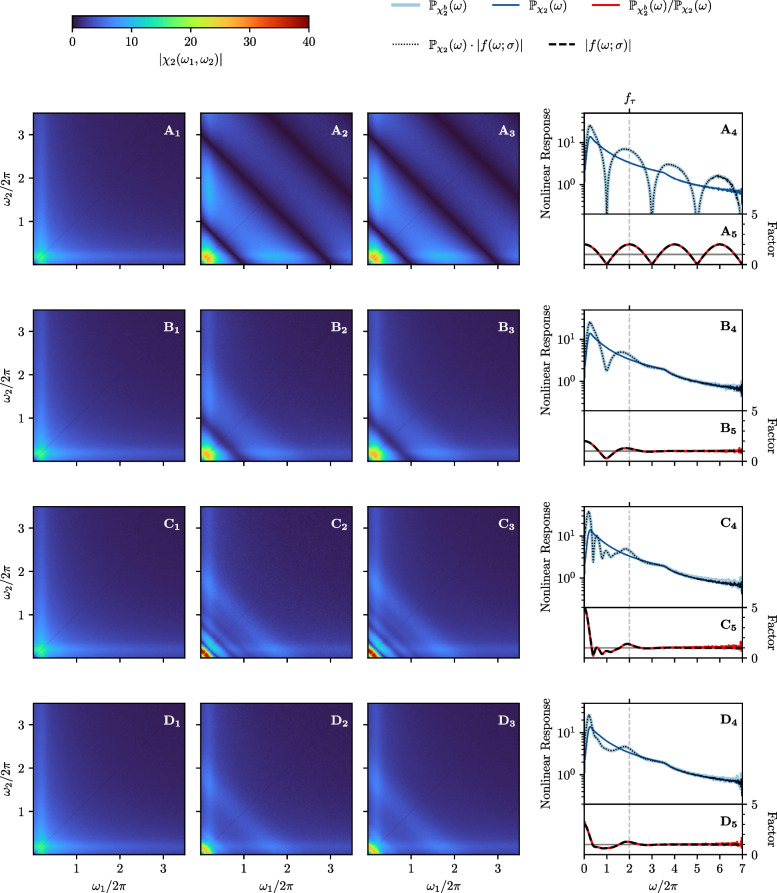
**LIF neuron: Effect of burst spikes on the nonlinear response function. A-D** same as in Fig. 2. [1]: Absolute value of the second-order susceptibility $\left| \chi _{2}(\omega _{1}, \omega _{2}) \right|$. [2]: Absolute value of the second-order burst susceptibility $\left| {\chi _{2}}^{b}(\omega _{1},\omega _{2}) \right|$. [3]: Absolute value of the theoretical prediction of the burst susceptibility, Eq. (29). [4]: Projection of the susceptibility (dark blue) and burst susceptibility (light blue) compared to the theoretical prediction of the projected burst susceptibility (black dotted line). [5]: Numerical (red) and analytical (black dashed line) estimation of the absolute value of the factor $\left| f(\omega ;\sigma )\right|$. Model parameters: same as in Fig. 4

### Nonlinear response function

Next, we want to verify our formulas for the nonlinear response function. The results are depicted in Fig. 5 and, as for the linear response, the panels A-D correspond to the application of the different burst algorithms in Fig. 2. We show the absolute value of the second-order susceptibility $\left| \chi _{2}(\omega _{1},\omega _{2}) \right|$ in the leftmost panel (index 1), the absolute value of the second-order burst-susceptibility $\left| {\chi }^{b}_{2}(\omega _{1},\omega _{2}) \right|$ in the middle left panel (index 2) and the absolute value of the theoretical prediction of the second-order burst-susceptibility, Eq. (29), in the middle right panel (index 3). The rightmost panel has the same structure as for the linear response: we show the projections (introduced in Eq. (30)) of the second-order susceptibility $\mathbb {P}_{\chi _{2}}(\omega )$ (dark blue) and burst susceptibility $\mathbb {P}_{{\chi }^{b}_{2}}(\omega )$ (light blue) as well as the projection of the theoretical prediction of the burst susceptibility (black dotted line) in the upper subpanel (index 4). In the lower subpanel (index 5) we compare again the numerical (red) and analytical (black dashed line) evaluation of the absolute value of the factor $\left| f_{\textrm{G}}(\omega ) \right|$; in 4 and 5 the frequency range is doubled, compared to the linear response, due to the projection to the sum of the frequency arguments, $\omega =\omega _{1} + \omega _{2}$.

For the nonlinear response function we make similar observations as for the linear response: along the diagonal, $\omega _{1} = \omega _{2}$, it is modulated by the same factor as the linear response function, only that the frequency argument of this factor is now $\omega = \omega _{1} + \omega _{2}$. As before, our numerical and analytical results are in excellent agreement.

If we just add one burst spike with a fixed delay $\tau$, we observe a perfectly periodic modulation of the nonlinear response with a maximum boost factor of two (Fig. 5$\text {A}_{1}$-$\text {A}_{5}$). We note that we would exactly obtain this increase of the nonlinear response at all frequencies if we would multiply the output spike train with a factor of two, i.e. place the additional burst spike at exactly the same spike time as the reference spike.

If we add a jitter to the single burst spike (Fig. 5$\text {B}_{1}$-$\text {B}_{5}$), the periodic modulation is damped with increasing frequency argument such that the added burst has practically no effect anymore at high frequencies. If we add four jittered burst spikes instead of one (Fig. 5$\text {C}_{1}$-$\text {C}_{5}$), we observe a higher boosting factor (maximum is five, reached at small frequencies) and still a pronounced damping effect. In addition, we find a subharmonic modulation with a period of $f_{\tau }/4$ and multiples of this frequency, which corresponds to the longer time scale of $4\tau$.

Finally, if we randomize the number of burst spikes (Fig. 5$\text {D}_{1}$-$\text {D}_{5}$), the subharmonic modulation is strongly diminished. What survives in this most realistic version of our burst algorithm is an amplification of the nonlinear response at small frequencies and at a sum frequency $\omega = 2\pi f_{\tau }$, a slight reduction for frequencies in between, and almost no effect for frequencies higher than the delay frequency $f_{\tau }$.

### Spike train power spectrum

**Fig. 6:**
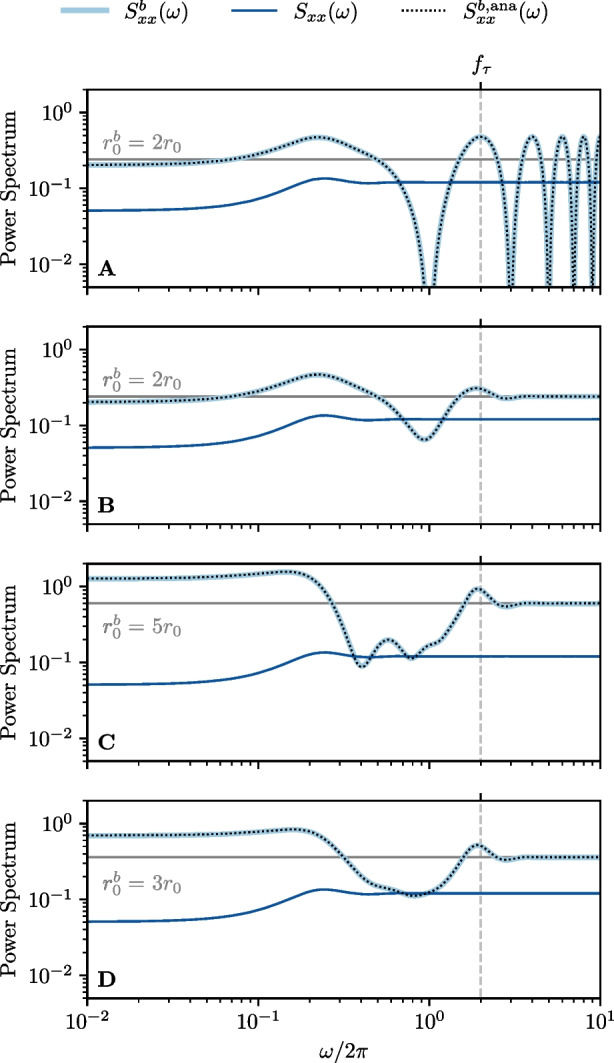
**LIF neuron: Effect of burst spikes on the spike train power spectrum. A-D** same as in Fig. 2. All panels: Numerical measurement of the power spectra of the spike train (dark blue) and burst spike train (light blue) compared to the theoretical prediction of the power spectrum of the burst spike train (black dotted line), Eq. (44). Model parameters: same as in Fig. 4

The last test for the LIF model is the spike-train power spectrum, and we show the results in Fig. 6. As for the response functions, the panel labels A-D correspond to the different burst algorithms illustrated in Fig. 2. We show the power spectrum of the reference spike train $S_{xx}(\omega )$ (dark blue), the power spectrum of the burst spike train ${S}^{b}_{xx}(\omega )$ (light blue) and the theoretical prediction of the burst-powerspectrum $S_{xx}^{b,\textrm{ana}}(\omega )$ (black dotted line) calculated from Eq. (44). We again find an excellent agreement of our numerical and analytical results for each different version of the burst algorithm.

In the special case of adding always one burst spike without a temporal jitter, i.e. $\sigma =0$ (panel A), we obtain as expected a periodic modulation of ${S}^{b}_{xx}(\omega )$ around the burst-firing rate ${r}^{b}_{0} = 2r_{0}$:

56 $$\begin{aligned} {S}^{b}_{xx}(\omega )&= 2S_{xx}(\omega ) \bigl [ 1 + \cos (\omega \tau ) \bigr ] \ . \end{aligned}$$

We note that this oscillation never ceases.

If we add a jitter, the oscillation is now damped with increasing frequency (panel B) and the power spectrum saturates at ${r}^{b}_{0}$. If we add four burst spikes instead of one (panel C), we increase the modulation amplitude and also add subharmonic modulation with frequency $f_{\tau }/4$. Finally, randomizing the number of burst spikes by drawing them from a uniform distribution (panel D), largely eliminates the subharmonic modulation and leaves an elevation of power at low frequencies and a pronounced peak at $f_{\tau }$ as the main effects of adding bursts on the spike-train power spectrum.

## Application to electroreceptor afferents

So far, we confirmed the derived relations between spectra of bursting and non-bursting spike trains for the LIF model; we expected this since we started from a non-bursting model and strictly followed our assumptions in applying the different versions of the burst-algorithm.

Starting from the observed effects of bursts on second-order susceptibility in electroreceptor afferents of weakly electric fish described in our companion paper (Barayeu et al., 2024) we here elaborated on the details of the burst algorithms and provide, based on the LIF neuron model, precise theoretical predictions. In this section we want to investigate to what extent our derived formulas can help us to understand features observed in the spectral statistics of bursty electroreceptor afferents from weakly electric fish. For this, we re-use data recorded in 75 bursting P-units (for details on the electrophysiological procedures see e.g. Grewe et al., 2017; Barayeu et al., 2023).

### Biological background and our approach to bursting

Weakly electric fish perceive their environment and communicate with conspecifics through an electrosense that comprises their electric organ, emitting the electric organ discharge (EOD), a precisely periodic signal, and thousands of electroreceptor cells that line the fish’s skin. One class of electroreceptor afferents are the probability units (P-units) that phase-lock to the fish’s own EOD and encode amplitude modulations of the self-generated electric field that are caused by other fish or objects in the environment (Scheich et al., 1973) by changes in their firing probability. Amplitude modulations in the form of broadband stimuli can be applied in the experiment, and long spike trains from the P-units in response to these signals can be recorded (Gussin et al., 2007; Grewe et al., 2017).

Typically, P-units already display a considerable spontaneous activity (i.e. in the absence of the stimulus) with firing rates between 50 Hz − 400 Hz, relatively high coefficient of variation (CV) of the ISI with values between ${0.2}{-}{0.9}$ (Grewe et al., 2017; Hladnik & Grewe, 2023), and multimodal ISI distributions with peaks at multiples of the EOD period $\tau _{\textrm{EOD}}$ (Chacron et al., 2000). A sizable fraction of P-units of the species Apternotus leptorhynchus displays bursting (Bastian, 1981a; Metzen et al., 2016), which becomes apparent by a bimodal modulation of the multimodal ISI density – the left peak of this bimodal envelope then defines the IBIs. Accordingly, bursts spikes are often defined by the criterion that the interval between them is smaller than 1.5 $\tau _{\textrm{EOD}}$ (Chan, 2005); here we occasionally chose 2.5 $\tau _{\textrm{EOD}}$ for specific cells if this better separates the two peaks in the envelope of the ISI distribution. The corresponding IBI distribution can then be approximated by a weighted sum of Gaussians, with mean values $\tau _{1}$, $\tau _{2}$ and standard deviations $\sigma _{1}$, $\sigma _{2}$, leading to the characteristic function

57 $$\begin{aligned} \varphi _{2\textrm{G}}(\omega )&= (1 - w) e^{i\omega \tau _{1} - \frac{1}{2} \omega ^{2} \sigma _{1}^{2}} + w e^{i\omega \tau _{2} - \frac{1}{2} \omega ^{2} \sigma _{2}^{2}} \ , \end{aligned}$$

where *w* is the small probability in the second Gaussian, i.e. for a IBI that is about twice the EOD period. We note that the mean value $\tau _{1}$ is close to but not necessarily identical to $\tau _{\textrm{EOD}}$.

We note that the spontaneous activity of P-units as well as their response to different kind of time-dependent stimuli has been modeled by different kinds of stochastic LIF models (endowed with an adaptation variable and a prefilter dynamics for the EOD inputs) (Barayeu et al., 2023). Also bursting in P-units has been the subject of some modeling attempts (Chan, 2005). Here, we do not aim at modeling bursting P-unit activity but instead pursue a statistical approach to the problem: We will remove the burst spikes from the *original spike train* (OST), defined by the criterion explained above, which yields the *reference spike train* (RST). By reintroducing burst spikes to the RST according to our burst algorithm and to the burst statistics of the specific cell, we obtain a new algorithmically created burst spike train (AST). By comparing the signal-transmission properties of these three types of spike trains, we can access the role of bursting in the encoding of time-dependent signals by P-units.

Specifically, we analyzed the spike trains of 75 bursty P-units of the species *Apteronotus leptorhynchus*, both during baseline activity and under stimulation with a random amplitude modulation. The spike trains and the respective EOD were recorded for $T_{\textrm{full}} = 10$s; the EOD-peak frequency, combined with a fine-tuning based on the phase-locked firing, provided us with the EOD period $\tau _{\textrm{EOD}}$ of the fish for this specific cell. To calculate the spectral statistics we split the full length into smaller time windows of $T = 0.5$s, resulting in a total of 20 trials for each cell.

According to the general recipe explained above, we extract for the OST the ISI density, can then apply the burst criterion, identify burst spikes and determine i) the burst-spike distribution and ii) the IBI distribution. We then remove the burst spikes to obtain the RST and finally add burst spikes again according to our algorithm (using the measured burst-spike and IBI distributions), to generate the AST.

**Fig. 7:**
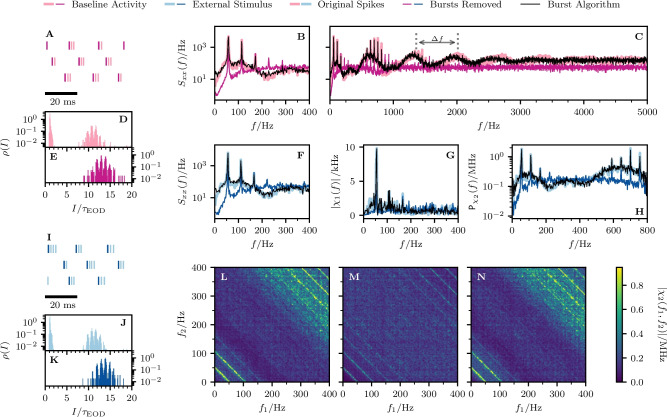
**Experimental data: Spectral statistics of a P-unit (cell**$\varvec{\mathcal {A}}$**). A-E** Baseline activity with OST (light purple) and RST (dark purple). **F-N** P-unit driven by an external stimulus with OST (light blue) and RST (dark blue). The black line shows the result for the AST in both cases (baseline activity and stimulus-driven). **A/I** Spike trains of 3 trials for the first 50 ms. **B/F** Power spectrum for $0< f < f_{\textrm{cut}}$. **C** Power spectrum for 0 Hz $< f<$ 5 kHz. **D-E**, **J-K** Interspike interval distributions. **G** Absolute value of the linear response susceptibility. **H** Projection of the nonlinear response. **L** Absolute value of the nonlinear response of the OST. **M** Absolute value of the nonlinear response of the RST. **N** Absolute value of the nonlinear response of the AST. The indicated period of the power spectrum’s undulation, $\Delta f \approx$ 655 Hz, is roughly given by the inverse of the fit parameter $\tau _{1}$ from Eq. (57), $1/\tau _{1} \approx$ 653 Hz

**Fig. 8:**
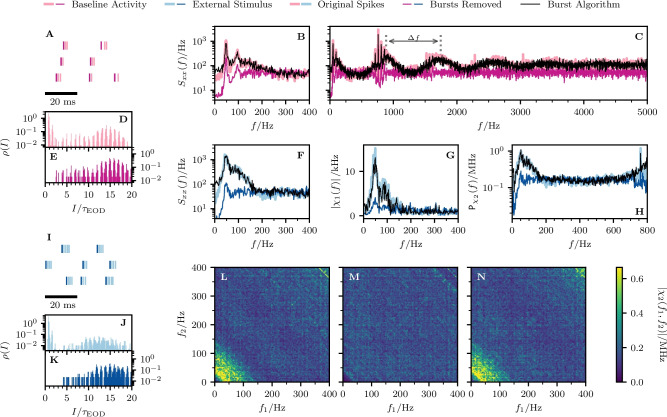
**Experimental data: Spectral statistics for a second cell (cell** $\varvec{\mathcal {B}}$**).** Same panels as in Fig. 7. The indicated period of the power spectrum’s ondulation, $\Delta f \approx$ 860 Hz, is roughly given by the inverse of the fit parameter $\tau _{1}$ from Eq. (57), $1/\tau _{1} \approx$ 864 Hz

**Fig. 9:**
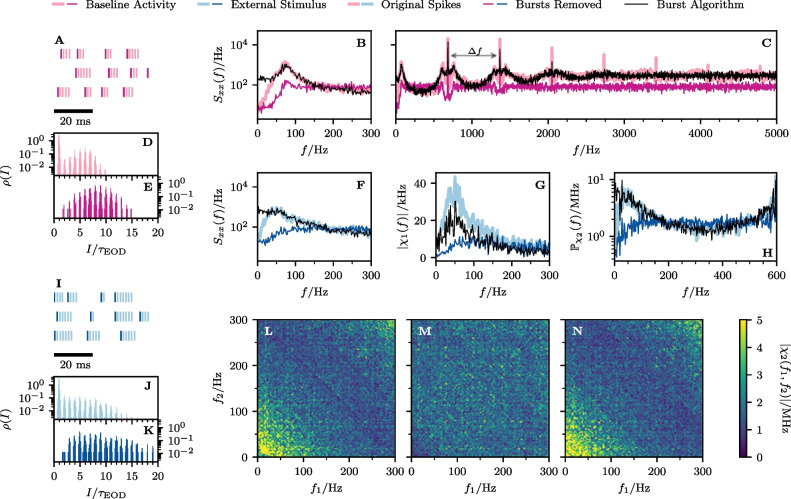
**Experimental data: Spectral statistics for a third cell (cell** $\varvec{\mathcal {C}}$**).** Same panels as in Fig. 7. The indicated period of the power spectrum’s ondulation, $\Delta f \approx$ 690 Hz, is roughly given by the inverse of the fit parameter $\tau _{1}$ from Eq. (57), $1/\tau _{1} \approx$ 693 Hz

### Statistics of three example cells

In the following we show the results for three example cells (Figs. 7, 8, and 9, respectively); the color scheme and panel organization is the same in all three figures: the baseline activity and the analyses performed on it is depicted in pink (panels A-E) while the data referring to the stimulus-driven situation are shown in blue (F-N). Results derived from the OST are depicted in light colors, those of the RST in dark colors, and those derived from the AST are shown in black. Panels A and I show the first 50ms of the first three trials for the baseline and stimulus-driven condition. We see that the spikes are arranged in *bursts* with a couple of spikes in short succession followed by a longer interval to the next burst. The number of spikes within a burst varies and also a single spike is possible; the burst-spike distributions are shown in the Appendix Section A. The first spike (the reference spike) within each burst is highlighted in dark colors; these spikes form the RST.

A clear manifestation of bursting can also be seen in the ISI distributions of cell $\mathcal {A}$ (Fig. 7D and J) which display a pronounced peak around the EOD period (corresponding to the IBIs) and peaks at multiples of $\tau _{\textrm{EOD}}$. The bimodal envelope structure of this distribution is a hallmark of bursting. If we remove the burst spikes, i.e. only consider the RST, the ISI distribution looses its first peak and the peaks at higher intervals are somewhat shifted to the right, both for the spontaneous firing (Fig. 7E) and under stimulation (K).

The power spectrum of the P-unit activity (thick light pink in Fig. 7B and C) has a complex structure with peaks at the firing rate of the RST, $r_{0} = {56.4}$ Hz (see Table 1), and its higher harmonics, a dip at low frequencies (a consequence of spike-frequency adaptation (Benda & Herz, 2003; Benda et al., 2005)), an overall modulation with a frequency roughly given by $1/\tau _{1}$ (see captions for the numerical values), and saturation in the high-frequency limit at ${r}^{b}_{0} = {160.7}$ Hz. On the contrary, the power spectrum of the RST (dark pink) is flat: it also displays peaks at $r_{0}$ and its higher harmonics at low frequencies but otherwise reaches the saturation level at $r_{0}$ fast. Remarkably, key features of the OST spectrum are reproduced by our statistical model, the AST: the sharp peaks and the overall spectral shape in an intermediate frequency range (50 Hz$-{400}$ Hz, see B), the high-frequency undulation (500 Hz$-{3000}$ Hz, see C), and the high-frequency limit. The only property not captured is the decreased power at low frequencies, an effect that is presumably due to spike-frequency adaptation and cannot be incorporated into our statistical model.

Adding a weak broadband stimulus with power between 0 Hz to 400 Hz does not change the power spectrum drastically in this cell (compare B and F), and the spectrum of the AST still shares key features with that of the OST (see agreement of light blue and black line). The linear response (susceptibility) of this cell and stimulation condition is particularly weak (see G) and characterized by a sharp peak around $r_{0}$ that sticks out of the noise floor. This is well reproduced by the AST (compare light blue and black lines), which is surprising because our statistical model does not take into account any effects of the stimulus on the bursting statistics. Even more surprising, frequency-dependent modulations of the *nonlinear* response of the OST (see L) that are absent for the RST (see M) are well reproduced by the AST (see N). The frequency modulation of the *nonlinear* response becomes best apparent by plotting the projection $\mathbb {P}$, Eq. (30), which illustrates how quantitatively accurate our statistical model can reproduce the effect of bursting on the nonlinear response (see H). We observe that by including burst spikes, the nonlinear response is boosted around the firing rate $f_{1} + f_{2} = r_{0}$ and around $1/\tau _{1}$, one of the characteristics of the IBI distribution (here determined in the presence of the stimulus).

**Table 1:** Spike statistics of the three example cells without (left) and with (right) a broadband stimulus being present

	Baseline activity					External stimulation				
Cells	${r}^{b}_{0}$ (Hz)	$r_{0}$ (Hz)	$\left\langle {N}^{b} \right\rangle$	$\tau _{1}^{-1}$ (Hz)	$f_{\textrm{EOD}}$ (Hz)	${r}^{b}_{0}$ (Hz)	$r_{0}$ (Hz)	$\left\langle {N}^{b} \right\rangle$	$\tau _{1}^{-1}$ (Hz)	$f_{\textrm{EOD}}$ (Hz)
$\mathcal {A}$	160.7	56.4	1.85	652.97	754.56	166.6	54.7	2.05	661.69	755.42
$\mathcal {B}$	112.7	53.5	1.11	863.92	760.45	199.7	49.7	3.02	885.97	760.11
$\mathcal {C}$	305.8	83.1	2.68	692.64	683.15	319.9	78.9	3.05	691.81	683.77

Turning to our second example, cell $\mathcal {B}$, shown in Fig. 8, we note several differences in the statistics. The multimodal ISI density of the OST has many more peaks and the IBIs also include values around $2\tau _{\textrm{EOD}}$. For the power spectrum under baseline conditions we make similar observations as for cell $\mathcal {A}$: the power spectrum of the OST displays a peak at the firing rate of the RST, $r_{0} = {53.5}$ Hz, and its second harmonic, a dip of power at low frequencies, an overall undulation with frequency $1/\tau _{1}$, and saturation in the high-frequency limit at ${r}^{b}_{0} =$ 112.7 Hz. Between 0 Hz and 400 Hz, the power spectrum of the RST displays the same peaks as the OST and like cell $\mathcal {A}$, the saturation level at $r_{0}$ is reached fast. Also, the AST for cell $\mathcal {B}$ nicely reproduces the key features of the spectrum except for the lowered power at low frequencies.

Unlike cell $\mathcal {A}$, we do not observe a submodulation of the power spectrum for frequencies $f < f_{\textrm{EOD}}$. Adding a weak stimulus we observe a clear impact of this driving input signal on the bursting properties of cell $\mathcal {B}$ (cf. ISI histograms in D and J) and the spectral statistics (cf. B and F). The linear response is generally stronger than that of cell $\mathcal {A}$ (cf. susceptibilities in G) whereas the nonlinear response is spectrally broadened and not as pronounced (cf. the broad stripes along the antidiagonal in L to the sharp lines in Fig. 7L). In all measures this cell is consistent with a higher level of output variability. The antidiagonal stripes of pronounced *nonlinear* response seen in the OST vanish for the RST (see M) but can be recovered by reintroduction of bursting in the AST (see N). All measures are well reproduced by the simulated AST spike trains with artificial (signal-unrelated) bursting (see H).

The third example cell (cell $\mathcal {C}$, statistics of interest in Fig. 9), apparently has an even larger output variability than cell $\mathcal {B}$: the peak around the spontaneous firing rate of the RST, that was sharp for cell $\mathcal {B}$ and very sharp for cell $\mathcal {A}$, is now rather broad (cf. B). Also, the power spectrum in the larger frequency range exhibits, in contrast to the previous cases, solely peaks at the EOD frequency and its multiples in combination with characteristic surrounding troughs of power and the familiar long-frequency undulation with period $1/\tau _1$. Stimulation with a broadband signal changes the ISI histogram of cell $\mathcal {C}$ drastically (cf. D and J for the OST and E and K for the RST) and, correspondingly, the power spectrum is additionally broadened (cf. F and B) and the susceptibility is strong (G). Furthermore, in line with the comparatively large variability of the cell, the nonlinear response is not pronounced and spectrally broad (cf. L-N and H). Considering only the RST, the observed structure of elevated nonlinear susceptibility vanishes. In contrast to cells $\mathcal {A}$ and $\mathcal {B}$, we see discrepancies between OST and AST for (i) the overall amplitude of the linear response (cf. light blue and black in G) and in the power spectrum at low frequencies (cf. B and F for $f < {40}$ Hz). We would like to stress that the nonlinear response, however, is largely captured by the AST.

From these three examples we can conclude that our (signal-unrelated) stochastic bursting algorithm can restore key frequency-dependent features in the spontaneous spiking as well as the linear and nonlinear responses; for some cells all features except for power spectra at low frequencies are quantitatively well captured (cells $\mathcal {A}$ and $\mathcal {B}$), additionally, for some cells the linear response seems to be stronger for the OST than for the AST (e.g. for cell $\mathcal {C}$). In all cases, the shaping of the nonlinear response is well captured for all cells.

**Fig. 10:**
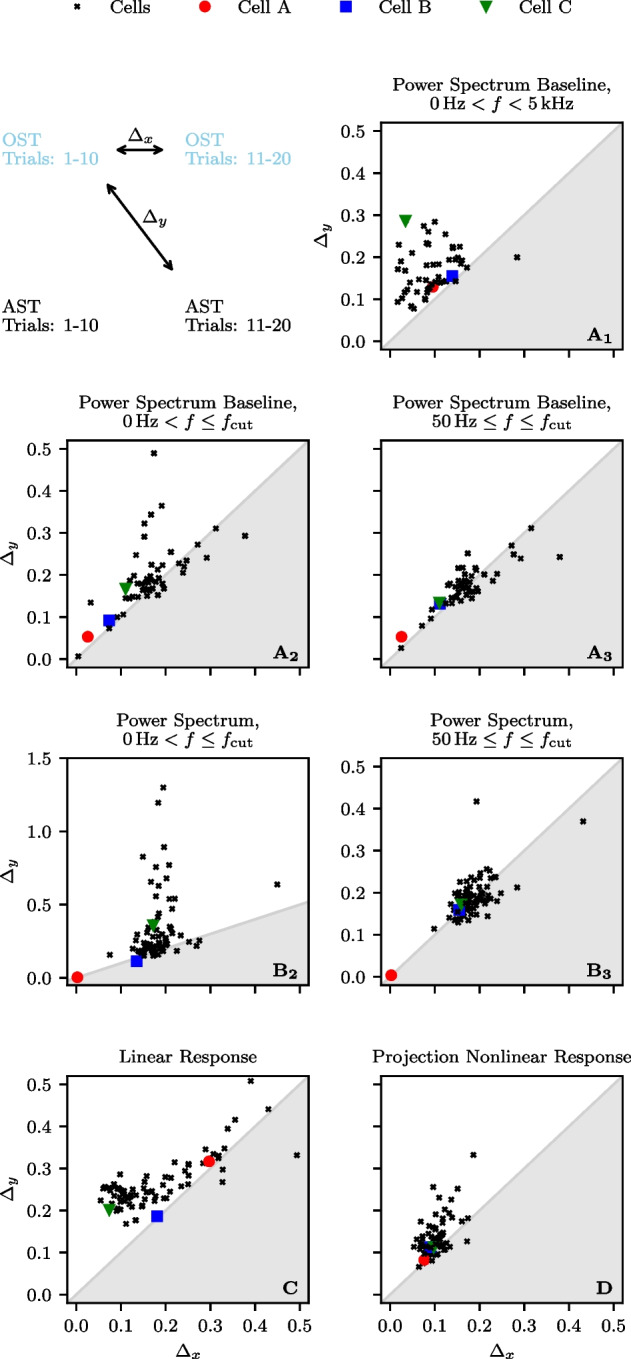
**Experimental data: Deviation to burst algorithm of 75 analyzed cells.** **A** Power spectrum in baseline activity. **B** Power spectrum with external stimulation. **C** Linear Response. **D** Projection nonlinear response. [1]: 0 Hz $< f<$ 5 kHz. [2]: $f \le f_{\textrm{cut}}$. [3]: 50 Hz $< f \le f_{\textrm{cut}}$. In all (labeled) panels we show the difference of the spectral statistics from the OST to the AST ($\Delta y$) and an estimation of the measurement noise ($\Delta x$) following the scheme in the top-left corner

In Fig. 10 we look at the differences between the spectral measures of OST and AST for all 75 bursting cells of our sample. Deviations between spectral statistics $F_1$ and $F_2(\omega )$ are quantified by a relative squared deviation integrated over a frequency band $[f_{\text {low}},f_{\text {up}}]$:

58 $$\begin{aligned} \Delta _{12} = \frac{\int \limits _{f_{\text {low}}}^{f_{\text {up}}} \text {d}{\omega }\ \bigl (F_{1}(\omega )-F_{2}(\omega )\bigr )^2}{\int \limits _{f_{\text {low}}}^{f_{\text {up}}} \text {d}{\omega }\ F_{1}^2(\omega )}. \end{aligned}$$

There are uncertainties in the estimates of the different spectral features that are due to the relatively small sample size. To cope with this unavoidable problem in experimental data, we always compare the differences in one spectral measure between OST and AST ($\Delta _y$) to the difference in the same spectral measure estimated from the first and the second half of the OST data ($\Delta _x$, see scheme in the top-left corner of Fig. 10).

If our burst algorithm captures all effects, the points $(\Delta _x, \Delta _y)$ should fall onto the diagonal. Points deviating from the diagonal indicate that bursting in P-units is more complicated than assumed in our burst algorithm. This is definitely the case for the majority of cells when considering the full power spectrum of the baseline activity (A$_1$) or the frequency range covered by the broadband stimulus in the absence of the stimulus (A$_2$) or the presence of the stimulus (B$_2$). Remarkably, if we take out the low-frequency range associated with spike-frequency adaptation ($< {50}$ Hz), the deviations become comparable to the noise floor, $\Delta _y \approx \Delta _x$, both for the baseline (A$_3$) and the driven activity (B$_3$). The linear susceptibility is systematically stronger for the OST than the AST (C). The difference between the nonlinear response of OST and AST seems to be overall small for about 90 % of the cells (D). We also indicate in all panels the three cells discussed above by colored symbols to give the reader some intuition about the quantification of the deviation. As can be expected, deviations for cells $\mathcal {A}$ and $\mathcal {B}$ are not significant (symbols are on the diagonal) whereas cell $\mathcal {C}$ is a representative of cells which deviate strongly from the diagonal (except for power spectra with an excluded low-frequency range).

**Fig. 11:**
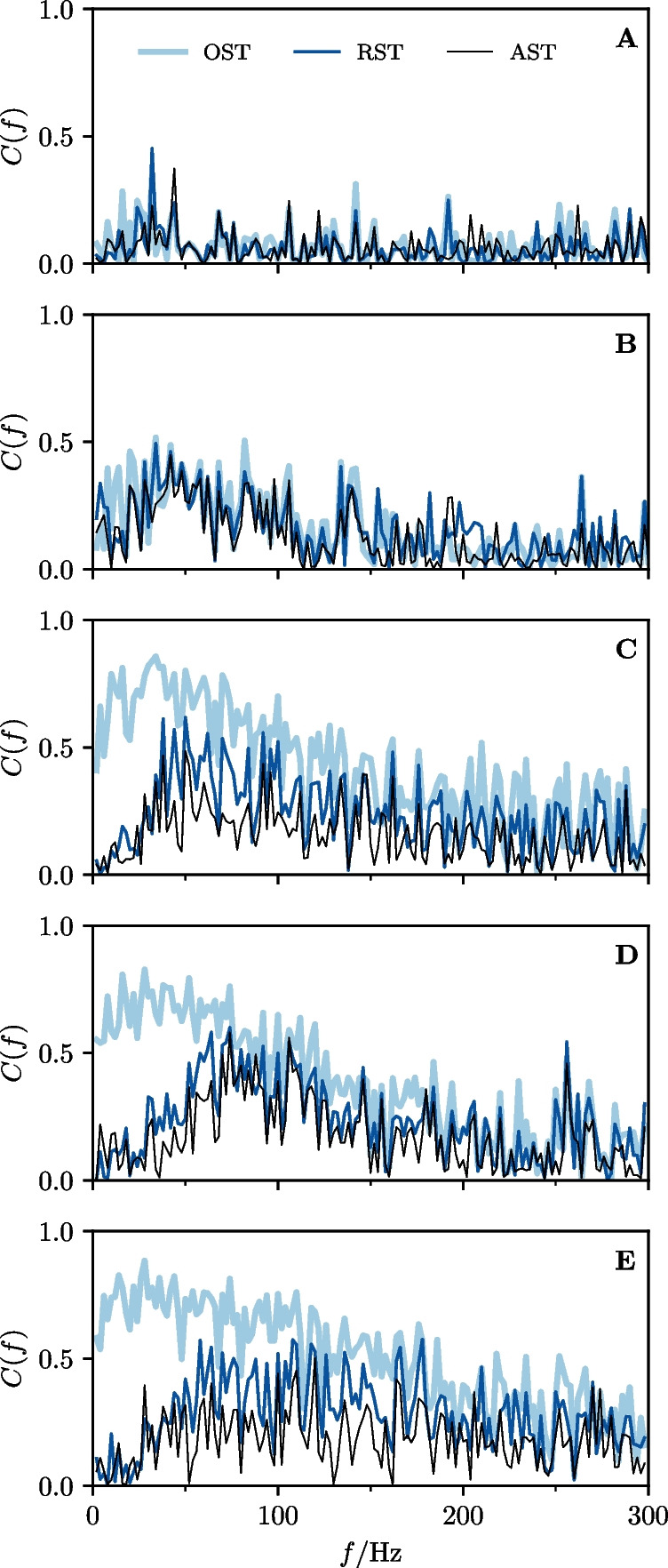
**Coherence functions for OST, RST and AST for five selected cells** **A** Cell $\mathcal {A}$, which is not significantly different from zero (cell has only little linear responsiveness); **B** Cell $\mathcal {B}$, which agrees largely for all three types of spike trains; **C** Cell $\mathcal {C}$, which shows strong enhancement of coherence by bursts and small reduction in the AST (black line below dark blue); **D** and **E**: two more cells that show a similar enhancement as cell $\mathcal {C}$, though in different frequency ranges

### Bursting affects coherence function and mutual information rate

We have seen that many features of the P-unit statistics can be well captured when we replace the true burst spikes by some artificially generated burst spikes, spikes that entirely ignore the driving stimulus and thus cannot improve but only degrade the information transmission. In a next step we compare the coherence functions for selected cells and mutual information rates for all 75 data sets for the OST, RST and AST.

We start with our three example cells from the previous subsection $\mathcal {A}$, $\mathcal {B}$, and $\mathcal {C}$. For cell $\mathcal {A}$, we saw already above that its linear response was very small. Accordingly, the coherence (Fig. 11A) is very low for most frequencies and in that also very similar for all three spike trains - here we do not find much of a difference between OST (thick light blue line), RST (blue line) and AST (black line). Similarly, for cell $\mathcal {B}$ (Fig. 11B) the coherence functions of the three types of spike trains are close to each other. We know from general theoretical considerations (cf. the inequality (51)) that the coherence of the AST has to be below that of the RST, and that is confirmed in the plots, however, the reduction of the coherence by adding burst spikes is not very pronounced.

Turning to cell $\mathcal {C}$ (Fig. 11C) and two more examples (Fig. 11D,E), the picture changes: the coherence of the OST is substantially higher than those of the RST and AST. Also, the coherence of the AST is reduced compared to that of the RST, as can be expected by the inequality (51). Both features are found for about 59 out of 75 data sets, i.e. the behavior seen in Fig. 11C-E is far more typical than that of cells $\mathcal {A}$ and $\mathcal {B}$.

**Fig. 12:**
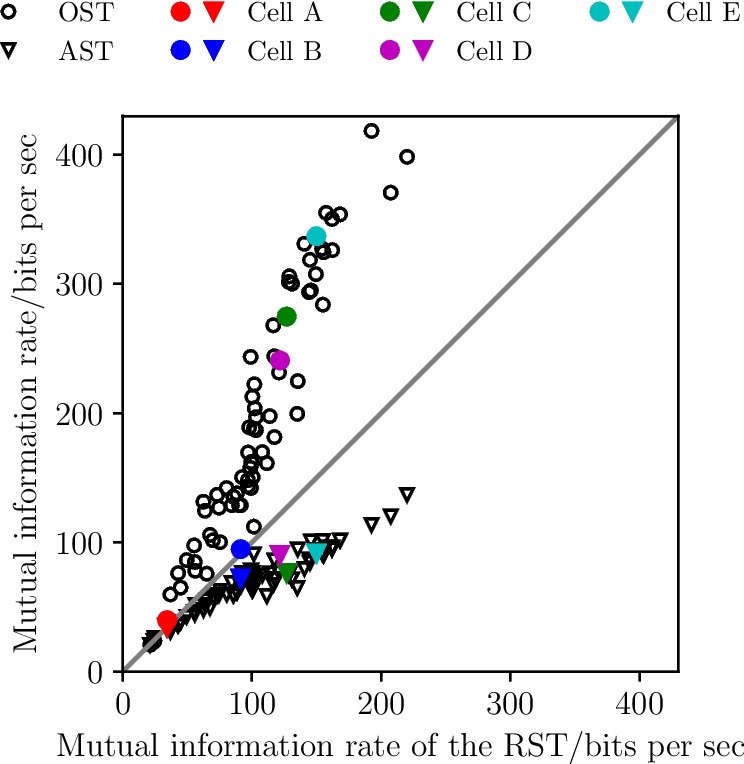
**Lower bound for mutual information rates of OST (circles) and of AST (triangles) vs that of the RST** Five selected cells from Fig. 11 are indicated by the respective colored symbols; lower bounds were computed via Eq. (10) from the coherence functions such as those shown in Fig. 11

Deviations in the coherence come about by a systematic underestimation of the susceptibility in the AST (entering the numerator of the coherence) and by the differences in the power spectrum (entering the denominator of the coherence). On their own, the deviations in the two spectral characteristics do not appear too strong (cf. the metastatistics discussed in the last subsection), their combined effect in the coherence, however, is significant and tells us that we miss something essential about the role of bursts when we replace true burst spikes with those coming from our simple algorithm. The discrepancy between the AST and real bursting becomes even more obvious when considering the lower bound estimate of the mutual information rate in Fig. 12. We plot the information rate for the OST over that of the RST of the corresponding cell. Almost all data points (shown as circles) are above the diagonal, i.e. the true bursts clearly increase the information transmission rate and the effect is most pronounced for those cells that have a high information rate. We also show the selected cells from Fig. 11 with colored circles and find, in line with the above discussion, cell $\mathcal {A}$ and $\mathcal {B}$ falling onto the diagonal, while the remaining cells deviate towards the top. Note that the range of information rates for the RST (the range on the *x* axis, which is about ${0}\,\text {bits}/\text {s}{-}{200}\,\text {bits}/\text {s}$) is about the range found for non-bursting cells (Wessel et al., 1996). This range is almost doubled by bursting - a pronounced effect.

In marked contrast, endowing the RST artificially with burst spikes in a signal-unrelated manner, i.e. generating the AST and considering its mutual information rate vs that of the RST, we do not increase the information transfer at all. Quite to the opposite, information rates are substantially reduced. This is particularly true for cells with higher values of the RST information rate: most of the triangles are below the diagonal.

## Summary and discussion

In this paper we have introduced a stochastic algorithm to endow a neural spike train with bursts that can reproduce important parts of the burst statistics extracted from experimental recordings. Most importantly, because of the simplicity of the algorithm of burst addition, we were able to find exact relations between the spectral statistics of the burst-free and the burst-endowed spike trains. This concerned not only the spontaneous activity but in particular the linear and weakly nonlinear response to time-dependent stimuli. In this way, we could access main effects of bursting on neural signal transmission.

After a detailed derivation of the relations between the statistics of burst-free and burst-endowed spike trains, we first tested our formulas for the spike trains of a stochastic leaky integrate-and-fire neuron, i.e. we added burst spikes to the spike trains of this model neuron, that was driven by a broad-band stimulus. In this setup we could expect perfect agreement because we exactly applied the burst algorithm as assumed in our derivations. Still, it became evident in this part of the study that adding a random number of jittered burst spikes to each reference spike shaped in a nontrivial frequency-dependent manner the statistics of the spontaneous firing (the spike-train power spectrum) as well as the linear and nonlinear response characteristics. In particular, in the nonlinear response certain frequency regions are strongly boosted while others are systematically suppressed.

In the next step we re-analyzed experimental spike trains from electroreceptor afferents, the P-units of weakly electric fish. We looked specifically at spike trains (the original spike trains, OST) that display substantial bursting, removed the bursts to obtain a reference spike train (RST) and added bursts again according to our algorithm. Most importantly, our stochastic algorithm does not incorporate the signal in any form (except for the spike times of the RST, that are of course affected by the signal). Despite this obvious shortcoming of the algorithm, the spike trains endowed with the artificial bursts, the AST, display spontaneous and response statistics that is very similar to those of the OST. This helps us to understand why bursting can, for instance, boost the nonlinear response of a neuron (Barayeu et al., 2024) although the signal may not even directly influence the bursting process.

The burst algorithm put forward here is certainly not the only one possible. Another version is obtained when a random number of burst spikes follow the reference spike at $t_k$ jittered around a lattice $t_k+n \tau$ with the same standard deviation around the respective lattice position for every burst spike. We have calculated the formulas for this algorithm as well, and this algorithm yields rather similar results for the spectral burst effects. One disadvantage of this version is, however, that the first intraburst interval (that still involved the reference spike time) is less variable than the following IBIs. This feature, in particular, makes it harder to apply this version of the stochastic burst algorithm to the experimental data. Another feature not included in our algorithm, is a systematic change in the IBI within the burst. Our analysis of the spontaneous activity reveals a dependence of the mean IBI vs its number within the burst: the IBIs tend to be longer towards the end of the burst (not shown). A more complicated statistical burst algorithm could take this effect into account by drawing IBIs from distributions with mean values increasing with the burst spike index. Here, we abstained from including such a systematic change of the mean IBI in the interest of analytical tractability.

If we think of the phase-locked firing of P-units that follow a rather precise external oscillation, the EOD, we might be tempted to generate bursts in manner that reproduces this feature, i.e. create burst spikes that are phase locked to the EOD. If one does this (in this case purely numerically) one obtains a spike train that might have spikes in very close proximity, i.e. with unrealistically short ISIs, which leads to artifacts in the spectral statistics. Our simple version in which burst spikes are added as a short renewal process takes the refractoriness of neurons much better into account and is also simpler to relate in its statistics to the reference spike train.

If we think not only of signal transmission but of the neural transmission of *information* we would like to see how bursting affects this. Upon closer inspection, it becomes quickly clear, though, that with our burst algorithms we cannot improve the information transmission. The reason is simply that adding burst spikes in a signal-unrelated manner only adds noise to the output of the neuron; this can never improve information transfer. Specifically, we could show that the coherence may be decreased but never increased by adding such burst spikes. This is in contrast to the different manifestations of stochastic resonance, for which a potentially beneficial noise is added on the input side of a spike-generating system and may lead to an enhancement of signal transmission (Longtin, 1993; Gammaitoni et al., 1998; McDonnell & Ward, 2011). However, it is unclear at the first glance whether the bursts exhibited by P-units add more noise or more signal to the output and can thus improve the information transmission.

Despite the success in the description of certain features of the spectral statistics of P-units, for a large fraction of these cells we observed also some quantitative discrepancies between the spike trains: the spike-train spectrum of the OST (both spontaneous and under a broad-band stimulus) displayed low power at low frequencies (below 50 Hz), whereas our spike train endowed with bursts, the AST, had much higher power in this range. Furthermore, the linear response was stronger for the OST than for the AST, i.e. the bursting is (moderately) modified by the stimulus signal. These moderate deviations have a strong combined effect in the measure of information transmission, the spectral coherence function, essentially the ratio of squared susceptibility (response function) and power spectrum: the original bursting spike train carries substantially more information on the time-dependent signal than the burst-free spike train (the RST) and even more so than the spike train endowed with stimulus-unrelated artificial burst spikes, the AST. The gain in information by burst spikes is most pronounced in the low-frequency range, i.e. for slow components of the Gaussian stimulus. Superficially, this may look similar to effects of bursting on the coherence function found in other electrosensory cells, specifically the pyramidal cells in the electrolateral line lobe of weakly electric fish, which has been studied by Oswald et al. (2004). We note, however, that these authors performed a different kind of data analysis, splitting the spike trains into a burst train (taking only the reference spikes of bursts for which ${N}^{b}_k\ge 1$) and a train of single spikes (reference spikes with ${N}^{b}_k=0$), omitting completely the role of the burst spikes that was in the focus of our study. This kind of approach provided insightful results on the role of burst spikes vs single spikes which were both important in the statistics of pyramidal cell’s firing. For the P-units studied here, the fraction of single spikes was small and thus the subdivision into burst reference spikes and single spikes (the sum of which does not account for the full spike train) would be less meaningful.

The fact that the original spike train has a significantly higher information rate than the RST shows the clear limitations of our burst algorithm. It also demonstrates specifically that bursting in P-units must originate in the spike generator (which is affected by the stimulus) and cannot be due to processes at the measuring site (the axon far from the spike generator). To study the role of bursting in P-units it is thus necessary to further study dynamical models of burst generation that can be affected by stimuli (accounting thus for the increased linear response) and for spike frequency adaptation (accounting for the reduced power in the spike-train spectrum). One disadvantage of such a approach is that a theory for the complex effects of bursting on spectral measures becomes very difficult. The statistical approach pursued in our paper gave at least a simple explanation for an essential number of those effects.

## Figures and Tables

**Fig. 13:**
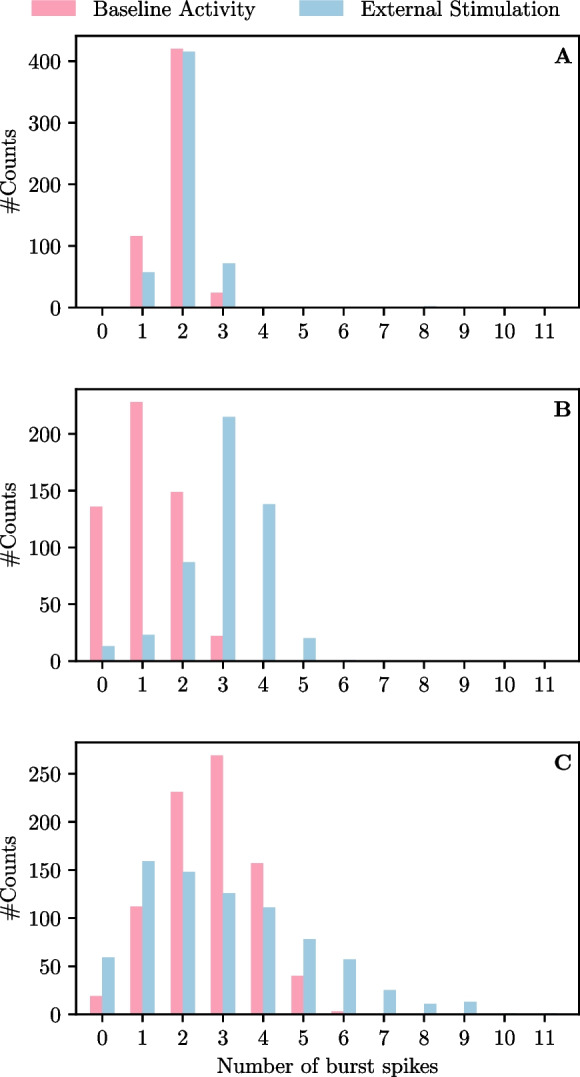
**Burst-spike distributions** under baseline condition (pink) and external stimulation (blue) of cell $\mathcal {A}$ (**A**), cell $\mathcal {B}$ (**B**) and cell $\mathcal {C}$ (**C**) discussed in Section 6.2. The number of burst spikes corresponds to the additional spikes to the first spike within a burst (reference spike), i.e. we have a single spike in the case of zero burst spikes

## Data Availability

No datasets were generated or analysed during the current study.

## A Burst-spike distributions of the three example cells

Figure 13 shows the burst-spike distributions of the cells $\mathcal {A}$, $\mathcal {B}$, and $\mathcal {C}$ discussed in Section 6.2. For once this plot illustrates the large variability of these statistics in the P-unit population. Furthermore, we also need these distributions for our burst-algorithm and state them here for completeness.

For cell $\mathcal {A}$ (panel A) we find similar distributions in presence (blue) and absence (pink) of the additional stimulus. This indicates that cell $\mathcal {A}$ responds very weakly to the external stimulus, which is in accordance with the observations discussed in Section 6.2.

Cell $\mathcal {B}$ (B) and $\mathcal {C}$ (C) show a clear impact of the external stimulus on the bursting statistics: the blue distributions are broadened compared to the pink ones. The stimulus causes the cell to fire larger bursts, i.e. a higher number of burst spikes. Comparing B and C this does not neccessarily mean that the distribution is simply shifted to a higher burst spike number (true for cell $\mathcal {B}$ but not for cell $\mathcal {C}$).
